# Cryogenic electron tomography reveals the mesoporous structure evolution during γ-Al_2_O_3_ supported Mo and MoNiP catalyst formation

**DOI:** 10.1039/d5cy01396h

**Published:** 2026-03-05

**Authors:** Jason M. J. J. Heinrichs, Angelina Evtushkova, Jovana Zečević, Thomas Weber, Heiner Friedrich, Emiel J. M. Hensen

**Affiliations:** a Laboratory of Inorganic Materials and Catalysis, Department of Chemical Engineering and Chemistry, Eindhoven University of Technology (TU/e) Den Dolech 2 5600 MB Eindhoven The Netherlands e.j.m.hensen@tue.nl; b Center for Multiscale Electron Microscopy, Department of Chemical Engineering and Chemistry, Eindhoven University of Technology (TU/e) Den Dolech 2 5600 MB Eindhoven The Netherlands; c Shell Global Solutions International B.V Grasweg 31 1031 HW Amsterdam The Netherlands; d Retired from Shell Global Solutions International B.V Grasweg 31 1031 HW Amsterdam The Netherlands; e Laboratory of Physical Chemistry, Department of Chemical Engineering and Chemistry, Eindhoven University of Technology (TU/e) Den Dolech 2 5600 MB Eindhoven The Netherlands

## Abstract

Heterogeneous catalysts play a central role in numerous industrially and societally relevant processes. Despite the widespread use, a detailed understanding of the support mesoporosity and its transformation during catalyst preparation remains incomplete. In this study, cryogenic electron tomography (cryo-ET) was utilized to resolve the 3D mesopore structure of γ-Al_2_O_3_ and to assess structural changes induced by oxidic Mo and MoNiP deposition and sulfidation during the preparation of hydrodesulfurization (HDS) catalysts. The intrinsic γ-Al_2_O_3_ surface and mesopore structure remained largely stable throughout calcination and sulfidation, although cryo-ET revealed subtle variations inaccessible to bulk characterization techniques. Oxidic Mo deposition introduced slight increases in tortuosity and surface corrugation, whereas oxidic MoNiP deposition induced minimal changes. Compared to the bare support, sulfidation of both Mo and MoNiP supported on γ-Al_2_O_3_ resulted in more tortuous mesopores and a more corrugated surface. Careful segmentation enabled separate analysis of γ-Al_2_O_3_ and MoS_2_ slabs, revealing that, for both catalysts, the γ-Al_2_O_3_ exhibited similar surface and mesopore modifications, and there were no significant differences in MoS_2_ slab morphology. Corrected Mo loadings derived from cryo-ET aligned with bulk measurements, validating the approach. These findings provide a comprehensive 3D perspective on mesopore stability during catalyst preparation and highlight the need for higher-resolution imaging and advanced 3D analysis to establish robust structure–function correlations.

## Abstract 2

Three-dimensional nanoscale analysis of mesopores provides insights into catalyst preparation by structurally assessing support structure, solids deposition, and activated metals.

## Graphical abstract

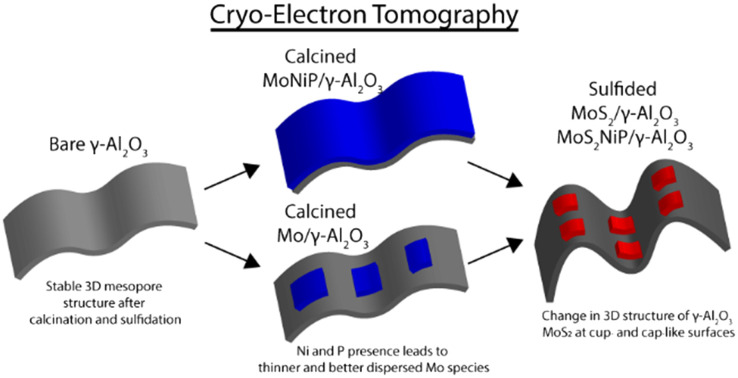

## Introduction

Ceramic high-surface area mesoporous materials are extensively utilized as support materials in heterogeneous catalysts, which makes them one of the most important classes of industrially employed materials. Heterogeneous catalysts consist of active particles, which are dispersed across and supported on a porous high-surface-area support material. The pore structure and topology of the support material significantly affect the distribution, shape, and accessibility of the deposited active constituents. Consequently, these support properties directly affect the performance of the active catalyst, including reactivity, selectivity, or stability.^1^

Prominent examples of industrial supports are high-surface-area variations of alumina, titania, zirconia, and silica, which can be optimized regarding porosity and other key properties, such as swelling or mechanical and thermal stability.^1^ The stability of support materials under operating conditions, such as high temperature and pressure, has been a subject of research interest since the 1990s.^2,3^ These research outcomes have largely contributed to the development of industrial catalysts with support pore structures and topologies engineered to the needs of the catalytic reaction. However, a detailed understanding of the mesopore-level structure and how it evolves during support manufacturing and catalyst preparation remains incomplete. Filling this gap would enable catalyst manufacturers to further optimize catalyst design and performance.

A typical method to prepare heterogeneous catalysts is incipient wetness impregnation (IWI). Here, a porous support material is impregnated with a solution that contains active constituents, followed by drying. Depending on the type of catalyst, subsequent heat-treatment steps under different gas compositions may follow. During drying, the dissolved species precipitate on the support surface according to the support's topology and its chemical interactions with the precipitates, potentially altering the intrinsic support network. Subsequent preparation steps, such as calcination or reduction, yield oxides or metals, respectively, and may lead to morphological changes in the deposited materials, which could alter the pore network of the catalyst. Additionally, calcination or reduction could change the intrinsic support pore network too, but this has not been thoroughly studied yet.

The processing steps during support manufacturing, catalyst preparation, and post-treatment steps can alter the three-dimensional (3D) surface and mesopore structure of the support and the catalyst. For example, the temperature used during the preparation of SBA-15 support particles determines the size of the (dis)ordered pores.^4^ Additionally, hydrothermal post-treatments can alter the mesopore connectivity and accessibility of ceria–zirconia oxides.^5,6^ Ultimately, the surface and pore structure of the support influences the particle morphology and location of the active particle, while the accessibility of the active particle is governed by the total catalyst pore network. Both aspects influence catalytic performance. Therefore, it is important to follow the support and catalyst surface and pore structure during catalyst preparation and post-treatment steps, which is the focus of this study.

We selected Mo(NiP) supported on disordered mesoporous γ-Al_2_O_3_ due to extensive prior research and the potential for future sustainable applications. Typically prepared through IWI followed by calcination and sulfidation, this catalyst class has been optimized over several decades primarily for hydrodesulfurization (HDS), the key industrial process for removing sulfur from crude oil. Ni-promoted Mo/γ-Al_2_O_3_ catalysts exhibit significantly higher HDS activity than their non-promoted counterparts, as Ni forms Ni–Mo–S species located at the edges of MoS_2_ slabs, thereby enhancing catalytic performance.^7,8^ Phosphorus is frequently added as a modifier because it partially passivates the acidic surface sites of γ-Al_2_O_3_ through the formation of aluminum phosphate species.^9–13^ This passivation weakens the strong metal–support interaction between Mo(Ni) species and γ-Al_2_O_3_. Moreover, P enhances the formation of type II MoS_2_ slabs, which possess higher intrinsic activity than type I slabs.^13–15^

A considerable body of research has explored various aspects of (promoted) MoS_2_/γ-Al_2_O_3_. This includes nanoscale visualization through transmission electron microscopy (TEM) and electron tomography (ET),^16–19^ characterization of porous architectures through X-ray computed tomography,^20,21^ studies on MoS_2_ slab morphology,^14,22,23^ investigations of pore and support effects on slab structure and catalytic performance,^24–28^ analyses of the liquid-impregnation process during catalyst preparation,^29,30^ studies of the effect of additives on (promoted) oxidic Mo catalysts,^15,31–36^ and the sulfidation process.^33,37–40^

More recently, MoS_2_-based catalysts have shown promising applications beyond HDS, including biomass upgrading,^38,41–44^ primarily hydrodeoxygenation (HDO),^38,41^ CO_2_ hydrogenation to methanol,^45^ and the water–gas shift reaction in sulfur-containing synthesis gas obtained by biomass gasification.^46,47^ γ-Al_2_O_3_ continues to be one of the most-widely used catalyst supports,^48^ raising important questions about the stability of γ-Al_2_O_3_, especially regarding its susceptibility to support dissolution during aqueous impregnation^1,48^ and subsequent preparation steps. Ultimately, a detailed understanding of how the pore structure of the support evolves throughout the catalyst preparation sequence is still lacking.

The pore size and structure of mesoporous materials can be probed using both indirect and direct methodologies. Indirect techniques such as Hg porosimetry and N_2_ physisorption enable estimation of the pore size distribution and pore volume by quantifying the amount of Hg intruded or N_2_ adsorbed.^49–52^ Additionally, N_2_ physisorption combined with a quantitative analytical method, such as liquid chromatography or differential scanning calorimetry (DSC), can be used to identify diffusion regimes^53^ or pore blocking phenomena^54^ at the bulk scale. While bulk characterization techniques provide insights into structural, electronic, and chemical properties of catalyst materials,^29,30,55–61^ ET offers direct three-dimensional visualization and quantitative analysis of catalyst morphology and mesopore architecture. For example, Arslan *et al.* (2008) demonstrated using ET that the support strongly influences the final catalyst morphology,^62^ and Nan *et al.* (2011) obtained nanospatial information on MoS_2_ slabs in a MoS_2_/SiO_2_ catalyst.^63^ However, to date, ET has not been applied to investigate the evolution of mesopore structure in γ-Al_2_O_3_-supported Mo catalysts during preparation.

Here, we study the pore structure of bare γ-Al_2_O_3_ during the different stages of IWI preparation, including calcination and sulfidation, as well as the structural changes that occur upon deposition of oxidic Mo or MoNiP precursors, followed by subsequent sulfidation. To this end, we employ cryogenic ET (cryo-ET), which provides 3D, nanometer-scale visualization of surface and pore structures. This approach enables both qualitative and quantitative analysis of pore morphology and, when catalytic phases can be distinguished from the support, the assessment of particle morphology and distribution within the porous network.^5,64–72^ Complementary characterization techniques, including N_2_ physisorption, synchrotron X-ray diffraction, and X-ray photoelectron spectroscopy, are used to validate and further contextualize the structural insights obtained from ET.

This study aims to develop a comprehensive structural understanding of Mo-based catalysts throughout their preparation. The insights generated are intended to support the rational design of Mo-based catalysts for emerging applications beyond well-established hydrotreating processes, for which the superior performance of MoNiP/γ-Al_2_O_3_ catalysts is already extensively documented. To this end, Mo/γ-Al_2_O_3_ and MoNiP/γ-Al_2_O_3_ are systematically compared to evaluate whether structural differences can be directly visualized using cryo-ET and to relate these nanoscale observations to established structural models.

Our results reveal that the intrinsic structural and electronic features of bare γ-Al_2_O_3_ are preserved after calcination and sulfidation, establishing a baseline for comparison of bare and Mo or MoNiP-filled γ-Al_2_O_3_. Initial observations indicate differences between Mo and MoNiP-filled γ-Al_2_O_3_ in terms of surface and pore structure descriptors, but notably, sulfidation results in similar surfaces, pores, and active phase descriptors.

## Experimental

### Catalyst preparation

A γ-Al_2_O_3_ support (Ketjen, CK300) was used for catalyst preparation. This material contains a disordered network of mesopores and has a H_2_O pore volume of 0.62 cm^3^ g^−1^. It is supplied as cylindrical extrudates with a diameter of 1 mm and a length of 3–10 mm. This support is produced commercially using a proprietary process that involves acid-peptization of pseudo-boehmite, extrusion of the resulting paste, followed by controlled drying, and calcination at intermediate temperatures. Catalysts were prepared by incipient wetness impregnation (IWI) on the γ-Al_2_O_3_ extrudates (Table 1). MoNiP/γ-Al_2_O_3_ was prepared using a metal salt solution obtained by mixing H_3_PO_4_ (85%) and MilliQ water (3 mL), followed by the addition and dissolution of MoO_3_ and NiCO_3_. Next, the solution was heated to 100 °C until a transparent green solution was obtained. To prepare Mo/γ-Al_2_O_3_, a metal salt solution was prepared by dissolving MoO_3_ in ammonium hydroxide (32% NH_3_ in H_2_O). MilliQ water was added to both precursor solutions to reach a total volume of 5 mL. These solutions were then used to impregnate the γ-Al_2_O_3_ extrudates. The impregnated extrudates were rotated on a roller bank in a sealed vial for ∼2 h, after which they were transferred to a ceramic crucible covered with perforated aluminium foil to allow drying in air at ambient temperature. The crucible was then placed in an oven overnight at 120 °C to ensure complete Solvent removal. The crucible was then placed in a calcination oven, where the material was heated to 400 °C for 2 h (Δ*T* = 6 °C min^−1^). Single-stage sulfidation was performed according to traditional model conditions to ensure simplicity, reproducibility and consistency with previously reported structural studies on similar catalysts.^20,22,73–75^ Sulfidation was performed in a stainless-steel reactor with an inner diameter of 4 mm. In detail, ∼75 mg of a crushed sieved fraction (75–125 μm) mixed with 200 mg SiC were loaded in the middle of the reactor and sulfided at ambient pressure for 2 h at 350 °C (Δ*T* = 6 °C min^−1^) in a 50 mL min^−1^ flow of 10 vol% H_2_S in H_2_. After sulfidation, the sample was transferred to a glovebox without air exposure until further characterization.

**Table 1:** Details regarding the IWI preparation of γ-Al_2_O_3_ supported Mo and MoNiP catalysts

Catalyst	MoO_3_ (g)	NiCO_3_ (g)	H_3_PO_4_ (g)	γ-Al_2_O_3_ (g)	Intended loading (wt%)		
					Mo	Ni	P
Mo/γ-Al_2_O_3_	1.42	—	—	8.06	10	—	—
MoNiP/γ-Al_2_O_3_	1.53	0.50	0.51	8.06	10	2.4	1.3

### (cryo-)TEM and ET sample preparation

TEM grids of 200 mesh Cu with a 6 nm thick continuous carbon layer were used for all measurements. Sample preparation involved gently crushing the catalyst extrudates into a fine powder, which was then dispersed in absolute ethanol. A small aliquot of the suspension was drop-cast onto a Au back-labelled TEM grid and allowed to dry, enabling the particles to adhere to the carbon layer. As a result, the particles remain attached to the carbon layer and thus ensuring stable imaging conditions.^76^ Preparation of sulfided TEM samples was performed entirely inside the glovebox to prevent exposure to air.

### (cryo-)TEM, ET, and STEM-EDX data acquisition

TEM images and tilt series were acquired on a Glacios cryo-TEM (TFS, Thermo Fisher Scientific) operated at 200 kV, equipped with a direct electron detector (FALCON 4i). For best results in (cryo-)TEM and (cryo-)ET, particles were selected with a thickness and width in the 100–300 nm range. 4 K tilt series, were acquired at a magnification of 92 k× (pixel size = 0.1535 nm) or 150 k× (pixel size = 0.0942 nm) within an angular range of −68° to 68° at increments of 1° and a nominal defocus of −1.0 μm (at 92 k×) or −0.2 μm (at 150 k×). At 92 k×, the dose rate was ∼5.1 e^−^ Å^−2^ image and the exposure time 0.3 s. At 150 k×, the dose rate was ∼9.0 e^−^ Å^−2^ · image and the exposure time 1.18 s. Potential beam damage was evaluated by acquiring images before and after the tilt series. No detrimental beam effects were observed at the used total dose.

STEM-EDX mapping was performed on a Titan Themis (FEI/TFS) operated at 300 kV and equipped with an XFlash 6 T|60 (Bruker) EDS detector. Elemental EDX maps were acquired at 40 k× with a beam convergence angle of 9.5 mrad and a camera length of 160 mm. Frames of approximately 450 × 450 pixels with a dwell time of 32 μs were recorded for 20 minutes. STEM-EDX maps were averaged by a [3 3] kernel prior to further analysis. K-edge quantification was performed with the Cliff-Lorimer method on Al, O, Mo, Ni, P, and S.

### 3D reconstruction and 3D quantitative analysis

Tilt-series alignment and tomographic reconstructions were performed in IMOD.^77–79^ In general, the aligned stacks (137 images with 1° tilt increments) were binned by a factor of 2. After binning, the final pixel size for the tilt series was 0.19 nm and 0.31 nm at 150 k× and 92 k×, respectively. To reconstruct the 3D volume, the simultaneous iterative reconstruction technique (SIRT) was used for 10 iterations only to prevent the fitting of noisy data, which is a consequence of the low dose used during acquisition. To reduce computational time during quantitative analysis, reconstructed volumes were trimmed to dimensions matching the imaged particle beforehand. The final volume was denoised by applying a 3D median filter with a kernel size of [3 3 3]. This filter preserves edge features present in the original 3D reconstruction.

Next, pixel classification (*i.e.*, segmentation) was performed through ILASTIK.^80,81^ Depending on the sample imaged, regions that could be identified as either background (*i.e.*, vacuum), γ-Al_2_O_3_, Mo/γ-Al_2_O_3_, MoNiP/γ-Al_2_O_3_, or MoS_2_ were manually annotated. The annotated regions were used to train a random forest classifier. This classifier groups pixels based on 3D-smoothed pixel intensity, edge filters, and texture descriptors. Each reconstruction was segmented by a unique random forest classifier model based on several input images originating from the same reconstruction.

After segmentation, a MATLAB script was used to clean the segmentation (*i.e.*, remove isolated voxels by majority operation, 3 iterations), after which the specific surface area (SSA) was calculated. The average strut width of the solid framework was obtained from the volumetric strut width distribution by multiplying the strut network skeleton by its distance map, both obtained through AMIRA3D. Next, morphological operations were performed to obtain the pore network and pore volume (PV). The pore network skeleton and distance map were obtained through AMIRA3D (TFS). The pore tortuosity (*i.e.*, the path between two pore nodes relative to the straight path) was determined *via* skeletonization in AMIRA3D. The average pore size was obtained from the volumetric pore size distribution (PSD) by multiplying the pore network skeleton by its distance map, also obtained through AMIRA3D. The shape index^82^ (*i.e.*, the local surface type: a cup-like (−1), a saddle-like (0), or a dome-like (1) surface) and curvedness^82^ (*i.e.*, bending degree of local surface type) of the isosurfaces of the solids were determined in AMIRA3D. Here, the variance of the shape index and curvature defines the corrugation of a surface. The higher the variance, the higher the corrugation, meaning more surface shape type and/or curvature strength fluctuations across a particle. Details regarding the estimation of the total corrugation per individual reconstructed particle are provided in the supplementary section S1. MoS_2_ slab descriptors, such as loading, thickness, nearest neighbor distance, perimeter, and interface, were quantified in MATLAB. The weight loading was calculated based on the total voxel volume of each component directly after segmentation. The thickness, nearest neighbor distance, perimeter, and interface were calculated after performing the majority operation. Finally, AMIRA3D was used to render 3D volumes of the reconstructions.

### Bulk characterization

N_2_ physisorption was used to determine the specific surface area (SSA), pore volume (PV), and pore size distribution (PSD) of the support or catalyst (50–200 mg; powder fraction 75–125 μm). Physisorption measurements were performed at −196 °C in a Micromeritics ASAP 2020 apparatus. Before measurements, all samples, except the sulfided samples, were pretreated in vacuum for 6 h at 100 °C. The pore size distribution (PSD) was determined using the Barrett–Joyner–Halenda (BJH) method, which was applied to the adsorption and desorption isotherms. To enhance accuracy, a Halsey thickness curve was employed, along with the Faas BJH correction, to account for potential deviations and improve the reliability of the pore size analysis. In this study, the PSD is the normalized volumetric contribution of a certain pore range to the total pore volume (dV/dW). It should not be mistaken for the derivative. The SSA was estimated by the Brunauer–Emmet–Teller (BET) method, and the PV was determined from the cumulative pore volume up to 15 nm in diameter, as derived from the BJH adsorption curve.

Inductively coupled plasma optical emission spectroscopy (ICP-OES) was used to analyze the elemental composition of the calcined catalysts. Measurements were carried out using a Spectro CIROS CCD Spectrometer. Typically, approximately 35 mg of the sample was dissolved in a mixture of 2 mL HNO_3_ (65%) and 4 mL of a 1 : 1 mixture of H_2_SO_4_ (97%) and H_2_O (1 : 1), with stirring to ensure complete dissolution.

Synchrotron X-ray diffraction (XRD) was used to evaluate the stability of γ-Al_2_O_3_ by determining its primary particle size after calcination and subsequent sulfidation. Measurements were carried out at the ID22 beamline at the ESRF synchrotron facility in Grenoble, France. Data were collected in transmission mode using an incident X-ray energy of 35 keV (*λ* = 0.354 Å). The scattered signal was detected with an Eigen2 XCdTe 2 M-W detector, preceded by 13 Si (111) crystals for optimal resolution. Samples were loaded into quartz capillaries (2 mm outer diameter, wall thickness 0.1 mm) and sealed by Beeswax to prevent contamination. Sulfided samples were prepared in a glovebox. The Scherrer equation was used to calculate the crystallite size (*i.e.*, primary particle size) of γ-Al_2_O_3_.

X-ray photoelectron spectroscopy (XPS) was used to analyze the surface composition, oxidation states, and sulfidation degree. To this end, a K-alpha XPS apparatus (Thermo Scientific) equipped with an aluminum anode (Al Kα = 1486.68) monochromatized X-ray source was used. All samples were finely ground and attached to double-sided carbon tape. Sulfided samples were prepared in a glovebox and transferred into the XPS apparatus *via* an airtight transport vessel. All spectra were recorded using a flood gun to reduce surface charging. By means of the software CasaXPS, a standard procedure involving calibration towards the Al 2p signal (74.1 eV) and Shirley background subtraction was performed on all spectral data. Fitting was performed with a symmetric pseudo-Voigt function (GL(30)).

Raman spectroscopy was employed to assess the structure, crystallinity, and coordination environment of the metal oxide precursor. Spectra were recorded using a Witec alpha 300 R confocal Raman microscope equipped with a 532 nm diode excitation source, a 1200 lines per mm grating (BLZ = 500 nm), and a charge-coupled device (CCD) was used. A ZEISS LD EC Epiplan-Neofluar Dic 50×/0.55 objective was used. Spectra of the powder samples were collected using 1200 accumulations with an integration time of 1 s per accumulation.

Ultraviolet-visible diffuse reflectance spectroscopy (UV-vis DRS) was used to investigate the coordination and aggregation states of the supported metal oxide phases. Spectra were collected from calcined powder samples (<75 μm) using a Thermo Scientific Evolution Pro UV-vis spectrophotometer. A spectral bandwidth of 1.5 nm, a measurement interval of 0.5 nm, and an integration time of 0.5 s were applied. BaSO_4_ served as the reflectance standard. The electronic edge energy (*E*_g_) of ligand-to-metal charge transfer transitions was determined from the intercept of the energy-axis and the tangent line of [*F*(*R*_∞_)*hv*]^2^*vs. hv* plot, where *F*(*R*_∞_) is the Kubelka–Munk function and *hv* is the photon energy.

Temperature-programmed reduction (TPR) was performed to study the reducibility of the samples. Experiments were performed on a Micromeritics AutoChem II instrument by loading 100 mg of powder (75–125 μm fraction) into a quartz U-tube reactor, positioned between two layers of quartz wool. Prior to analysis, the samples were pretreated at 200 °C for 1 h in a flow of 50 mL min^−1^ He. TPR profiles were recorded by heating the sample from 40 °C to 900 °C at a 5 °C min^−1^ rate in a 50 mL min^−1^ flow of 4 vol% H_2_ in N_2_. H_2_ consumption was monitored by a thermal conductivity detector (TCD), which was calibrated with an AgO reference.

## Results and discussion

### Bare γ-Al_2_O_3_ supports (untreated, calcined, and sulfided)

To determine whether the pore structure of the γ-Al_2_O_3_ support changes during catalyst preparation, it is essential to investigate the bare support prior to and after each synthesis step, namely the untreated, calcined, and sulfided states. Over years of storage and handling, untreated γ-Al_2_O_3_ is exposed to air and humidity, resulting in re-hydroxylation of the surface and equilibration with ambient conditions. To refresh the support surface and thereby remove physisorbed water, regenerate hydroxyl groups, and eliminate contaminants, the untreated γ-Al_2_O_3_ was calcined prior to use. Accordingly, we deemed it necessary to evaluate the stability of the γ-Al_2_O_3_ mesopore structure at the nanoscale after both the calcination and sulfidation steps. Ideally, many datasets would be averaged to yield a statistically robust distribution of quantitative pore structure descriptors. However, such extensive data collection is currently impractical with cryo-ET due to substantial time required for acquisition, processing, and analysis. To balance experimental feasibility with statistical relevance, each sample state was therefore imaged in triplicate.

**Fig. 1:**
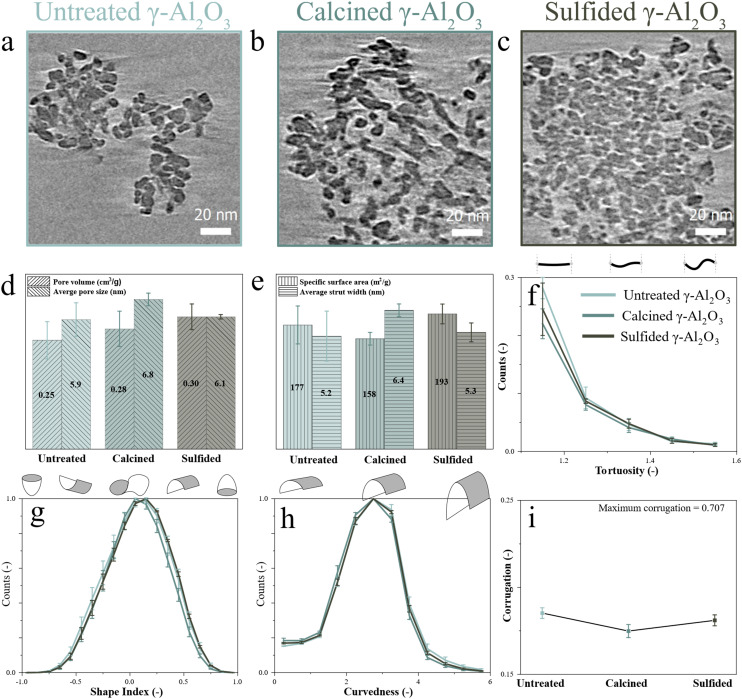
Quantitative ET showing numerical cross sections (thickness = 0.31 nm) through cryo-ET reconstructions of bare γ-Al_2_O_3_ supports in (a) untreated, (b) calcined, and (c) sulfided state (each triplo). Quantitative analysis of the surface and pore network showing the averaged values for: (d) PV and average pore size; (e) SSA and average strut width; (f) tortuosity; (g) shape index, (h) curvedness, and (i) corrugation.

Previous work has shown that the untreated bare γ-Al_2_O_3_ used in this study exhibits regions with different pore structures.^83^Fig. 1(a–c) displays numerical cross sections through the reconstructions of untreated, calcined, and sulfided γ-Al_2_O_3_ support particles. Additionally, numerical cross sections from the top, middle, and bottom regions of all reconstructed particles are shown in SI section S2. Among the states, the observed particles varied in size, which can be attributed to the crushing of the extrudates during TEM sample preparation. Variations in the packing density and size of the fused γ-Al_2_O_3_ primary particles were observed, which are expected to lead to differences in pore structure descriptors. These variations are not attributed to calcination or sulfidation, as they were observed in the pristine material too (Fig. S2.1). No other visual differences were observed between the states, while quantifying the cryo-ET data can provide a much more detailed description of the mesopore network and possible effects of calcination and sulfidation.

To obtain quantitative insights into the γ-Al_2_O_3_ pore structure, the support structure was isolated (segmented) from the reconstructed cryo-ET data, resulting in a binary 3D dataset for detailed analysis (Fig. S2.4). For each state, surface and pore structure descriptors of three unique segmented 3D datasets, representing individual γ-Al_2_O_3_ support particles, were quantified and averaged to assess the preservation of the support. Additionally, bulk characterization techniques were employed to determine the preservation of the main physicochemical properties of the γ-Al_2_O_3_ throughout the preparation process.

The bulk pore structure was estimated by N_2_ physisorption. As shown in Fig. S3.1(a), the untreated, calcined, and sulfided γ-Al_2_O_3_ samples exhibit a type IV(a) isotherm, characteristic of mesoporous materials_._^50^ The adsorption–desorption branches and the associated hysteresis loops indicate that the overall pore structures of the three γ-Al_2_O_3_ states appear very similar. In contrast, the averaged cryo-ET data, shown in Fig. 1(d and e), reveal pronounced differences in pore volume (PV), pore size (distribution in Fig. S2.5), specific surface area (SSA), and strut width among the states. These variations corroborate the qualitative differences evident from visual inspection of the reconstructions.

For each state, the PV derived from cryo-ET (Fig. 1(d)) is significantly smaller than the PV derived from N_2_ physisorption analysis (Fig. S3.1(b)). This discrepancy likely arises from fracturing of the structure along the largest pores during sample preparation, where grinding may preferentially open wide pores. As a result, these larger pores are no longer enclosed volumes and are therefore not captured in the PV determination by cryo-ET. The PV measured by cryo-ET remains essentially constant following calcination and sulfidation. For each state, the average pore size estimated by cryo-ET (Fig. 1(d)) is in good agreement with the desorption BJH pore size distributions (Fig. S3.1(d)). Additionally, the SSA calculated from cryo-ET (Fig. 1(e)) matches well the BET results (Fig. S3.1(b)). Finally, for each state, the average strut width, measured by cryo-ET (Fig. 1(e)), showed variations from 4.0 to 6.7 nm. This aligns with the presence of a range of primary γ-Al_2_O_3_ platelet sizes at every state. The application of the Scherrer equation to the synchrotron XRD data revealed crystallite sizes ranging from 4.2 to 8.4 nm for the untreated and calcined γ-Al_2_O_3_ and 4.0 to 8.4 nm for the sulfided γ-Al_2_O_3_ (Fig. S3.3 and Table S3.2). These results suggest that the primary γ-Al_2_O_3_ platelets remain stable during calcination and sulfidation.

In addition, cryo-ET enabled a more detailed analysis of structural features that were not directly accessible by bulk characterization. In Fig. 1(f–i), the tortuosity, shape index, curvedness, and corrugation of untreated, calcined, and sulfided γ-Al_2_O_3_ are shown. These descriptors exhibit only minor variations with overlapping error ranges. Therefore, the structural features of bare γ-Al_2_O_3_ appear to be preserved following calcination and sulfidation.

Based on the combined assessment of the cryo-ET data and bulk characterization results, γ-Al_2_O_3_ appears structurally stable after both calcination and sulfidation. The observed variations in surface and pore descriptors are therefore more attributed to intrinsic local heterogeneity of the support, heterogeneity that cryo-ET is uniquely sensitive to, rather than from systematic modification of the γ-Al_2_O_3_ framework. Moreover, the combined set of descriptors obtained for each particle can be used to construct a structural model of the pore architecture, which can be used to illustrate the relation among individual descriptors (SI section S4). To establish a reference for the intrinsic structural heterogeneity of the bare γ-Al_2_O_3_, the average values from the untreated, calcined, and sulfided datasets, comprising a total of 9 reconstructed particles, are used as a baseline for comparison with Mo or MoNiP-containing γ-Al_2_O_3_ samples, which will be discussed in the next section.

### γ-Al_2_O_3_ supported Mo or MoNiP after calcination and sulfidation

This section examines the changes in surface and pore structure following the deposition of oxidic Mo or MoNiP on γ-Al_2_O_3_ supports and subsequent sulfidation. To ensure a fair comparison, the intended Mo weight loading for both Mo/γ-Al_2_O_3_ and MoNiP/γ-Al_2_O_3_ is 10 wt%. The actual Mo loadings of the calcined samples were characterized using ICP-OES, XPS, and STEM-EDX, with results shown in the SI (Tables S3.5 and S3.6). Moreover, as shown in Fig. S3.7(a) and S3.8(a), STEM-EDX reveals a uniform sub-micron-level distribution of the calcined Mo in Mo/γ-Al_2_O_3_ and of calcined Mo, Ni, P in MoNiP/γ-Al_2_O_3_.

**Fig. 2:**
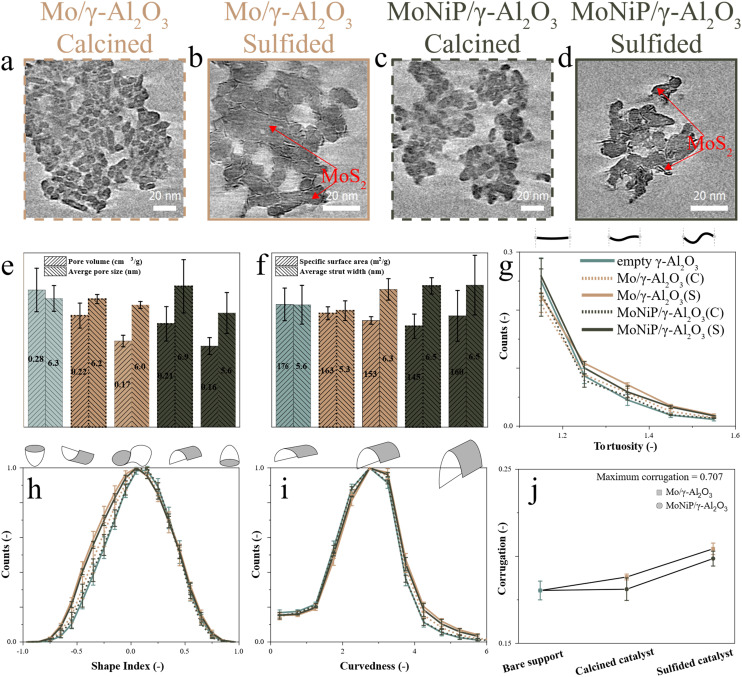
Quantitative ET showing numerical cross sections ((a and c) thickness = 0.31 nm; (b and d) thickness = 0.19 nm) through cryo-ET reconstruction of calcined (a and c) and sulfided (b and d) Mo/γ-Al_2_O_3_ and MoNiP/γ-Al_2_O_3_. An enlarged image of (a–d) is shown in Fig. S5.1(a–d). The formed MoS_2_ particles are indicated by red arrows. Quantitative analysis of the surface and pore network showing: (e) PV and average pore size; (f) SSA and average strut width; (g) tortuosity; (h) shape index, (i) curvedness; and (j) surface corrugation.

Numerical cross sections of calcined and sulfided Mo/γ-Al_2_O_3_ are shown in Fig. 2(a and b), with an enlarged view in Fig. S5.1(a and b). Cross sections from the top, middle, and bottom regions of all reconstructed Mo/γ-Al_2_O_3_ particles are shown in Fig. S5.2 and S5.3. The calcined Mo/γ-Al_2_O_3_ particles exhibit a similar structure to the bare γ-Al_2_O_3_, where the fused primary particles form the porous backbone. The absence of features that could be associated with segregated Mo oxide particles indicates that oxidic Mo is uniformly dispersed at the nanometer scale across the γ-Al_2_O_3_ surface. Due to this uniform distribution, insufficient contrast existed between the two materials for the oxidic Mo to be identified. After sulfidation, the structure of the primary γ-Al_2_O_3_ particles changed, and oxidic Mo transformed into MoS_2_ slabs visible on the surface of the γ-Al_2_O_3_ (Fig. 2(a and c)). A variety of basal and edge plane connections between the slabs and γ-Al_2_O_3_, combined with minimal slab stacking, were observed. Similar observations were made for the numerical cross sections of calcined and sulfided MoNiP/γ-Al_2_O_3_, shown in Fig. 2(c and d), with an enlarged view in Fig. S5.1(c and d) and cross sections from the top, middle, and bottom regions of all reconstructed MoNiP/γ-Al_2_O_3_ particles in Fig. S5.4 and S5.5. How and to what extent the deposition of calcined Mo or MoNiP and subsequent sulfidation change the pore structure with respect to the bare γ-Al_2_O_3_ will be discussed in the next section.

To extract surface and mesopore structure descriptors inaccessible through visual interpretation alone, the solid material depicted in Fig. 2(a–d) was segmented. Slices of the segmented volumes are shown in Fig. S5.6. Descriptors were extracted for three distinct Mo/γ-Al_2_O_3_ and MoNiP/γ-Al_2_O_3_ particles, with results averaged for the calcined and sulfided states, and compared to initially bare γ-Al_2_O_3_. Bulk characterization techniques were employed to assess the primary physicochemical properties following solids deposition and sulfidation.

The effect of deposition of both oxidic Mo and MoNiP (*i.e.*, the calcined state) on the γ-Al_2_O_3_ surface, as well as the following sulfidation, resulted in different surface and mesopore structures compared to the initially bare γ-Al_2_O_3_. As shown in Fig. 2(e), a similar decrease in PV occurred after the deposition of oxidic Mo or MoNiP and sulfidation. Additionally, the bulk PV decreased similarly after solids deposition (Fig. S3.1 and Table S3.1), suggesting preferential deposition of solids in pores ranging from 2 to 15 nm, which are more prevalent in cryo-ET specimens than in the bulk powder. Notably, cryo-ET suggests that after Mo or MoNiP deposition and sulfidation, the average pore size remained within the initial range determined for bare γ-Al_2_O_3_. This is also observed in the bulk, as BJH analysis of the adsorption and desorption branches, shown in Fig. S3.1(c and d), does not reveal a significant decrease in the PSD after solids deposition or sulfidation.

In Fig. 2(f), the SSA and average strut width after solids deposition and subsequent sulfidation are depicted. The subtle trends in SSA resemble those observed in bulk (Fig. S3.1 and Table S3.1), suggesting that the SSA is primarily determined by the mesopores. Moreover, both cryo-ET and bulk show a sequential SSA decrease after solids deposition and sulfidation for Mo/γ-Al_2_O_3_. In contrast, for MoNiP/γ-Al_2_O_3_, the SSA decreases after solids deposition but shows a slight increase after sulfidation. In this study, it is not possible to unambiguously assign the observed increase in SSA for Mo/γ-Al_2_O_3_ and the decrease for MoNiP/γ-Al_2_O_3_ to specific molecular-level mechanisms. Nevertheless, these trends are likely linked to the broader structural and compositional changes occurring during sulfidation. Formation of MoS_2_ involves replacement of O by S, which occupies a larger atomic volume than O in the oxidic Mo species, and visual inspection reveals accompanying changes to the γ-Al_2_O_3_ framework, altering its pore and surface structure. The presence of Ni and P is expected to influence MoS_2_ morphology and may also affect the γ-Al_2_O_3_ surface after sulfidation compared with Mo-only catalysts. The average strut width, found through cryo-ET, remains within the range observed for the bare γ-Al_2_O_3_, implying that deposition of Mo or MoNiP does not substantially change the average strut width.

Cryo-ET descriptors inaccessible by bulk characterization revealed distinct differences in the surface and mesopore structure after calcination and sulfidation of the bare γ-Al_2_O_3_, Mo/γ-Al_2_O_3_, and MoNiP/γ-Al_2_O_3_. During calcination, aggregated molybdate structures are expected to form on the γ-Al_2_O_3_ surface.^84^Fig. 2(g–j) show that, compared to the bare γ-Al_2_O_3_, the Mo/γ-Al_2_O_3_ exhibits a more tortuous pore system (*i.e.*, higher tortuosity) and a more corrugated surface (*i.e.*, broadened shape index and curvedness distribution), suggesting the formation of topographically prominent Mo domains. In contrast, the tortuosity and corrugation of MoNiP/γ-Al_2_O_3_ are similar to the bare γ-Al_2_O_3_, suggesting that the oxidic MoNiP is distributed as a thin layer that does not alter the pores and surface. This interpretation is consistent with UV-vis DRS, suggesting smaller and thinner Mo aggregates in MoNiP/γ-Al_2_O_3_ (Fig. S3.5), and Raman displaying more tetrahedrally coordinated (isolated) Mo species in Mo/γ-Al_2_O_3_ (Fig. S3.6).

H_2_-TPR data (Fig. S3.7) reveals that Mo/γ-Al_2_O_3_ undergoes reduction at higher temperatures and exhibits a pronounced high-temperature bulk-reduction feature, consistent with strong stabilization of Mo–O–Al species. In contrast, MoNiP/γ-Al_2_O_3_ shows a significant shift of the main reduction peak to lower temperatures and the disappearance of the bulk-reduction signal. This behavior reflects a markedly weakened effective metal–support interaction, arising from suppression of strong Mo–O–Al bonding and the formation of thin Mo–O–P networks near Ni. The presence of Ni and P enhances hydrogen accessibility to Mo species and thus facilitates reduction.

In future work, it will be important to gain insights into the exact location of deposited oxidic species. This requires deposits to be distinguished from the support, which was not possible for the combination of Mo and γ-Al_2_O_3_ in this study. There are various routes to accomplish this, such as four-dimensional (4D) scanning TEM, integrated differential phase contrast (iDPC) - STEM, or segmentation through deep learning.^70,85,86^

During the sulfidation step, oxidic Mo converts to MoS_2_, which is the active catalyst phase. Interestingly, after sulfidation, the pore morphologies of Mo/γ-Al_2_O_3_ and MoNiP/γ-Al_2_O_3_ are quite similar yet different from the bare γ-Al_2_O_3_ (Fig. 2(g–j)). During sulfidation, immobilized MoO_*x*_ species are transformed into mobile MoS_*x*_ domains, which aggregate and grow into MoS_2_ slabs.^84^ The resulting MoS_2_ particles affect the pore structure of both MoNiP/γ-Al_2_O_3_ and Mo/γ-Al_2_O_3_ similarly, suggesting that the MoS_2_ particles occupy comparable positions on the γ-Al_2_O_3_ surface.

### Direct quantification of isolated γ-Al_2_O_3_ and MoS_2_ slabs after sulfidation

**Fig. 3:**
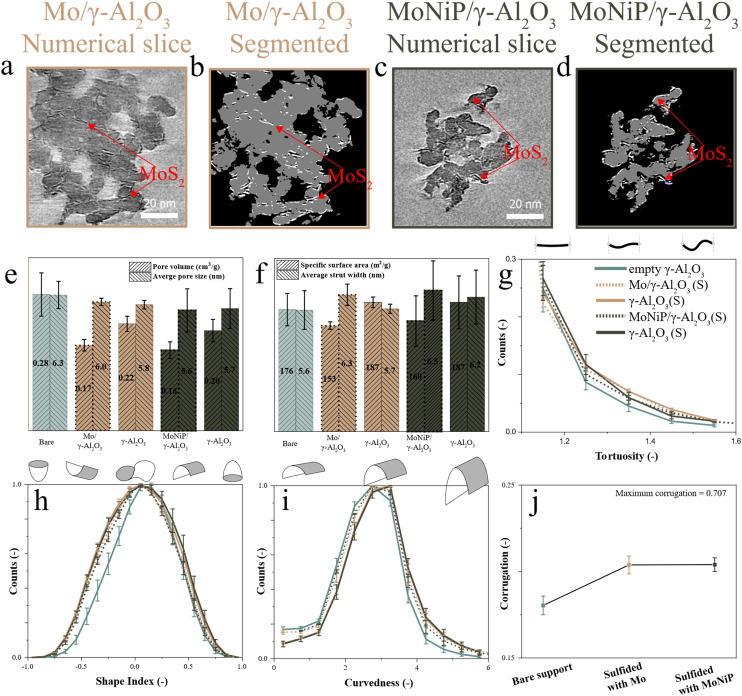
Quantitative ET showing numerical cross sections (thickness = 0.19 nm) and corresponding segmented cross sections of sulfided γ-Al_2_O_3_ segmented from the Mo/γ-Al_2_O_3_ (a and b) and MoNiP/γ-Al_2_O_3_ (c and d). The γ-Al_2_O_3_ is compared to the sulfided full solids Mo/γ-Al_2_O_3_ and MoNiP/γ-Al_2_O_3_. The formed MoS_2_ particles are indicated by red arrows. Quantitative analysis of the surface and pore network showing: (e) PV and average pore size; (f) SSA and average strut width; (g) tortuosity; (h) shape index, (i) curvedness; and (j) surface corrugation.

The previous section showed that the deposition of oxidic Mo or MoNiP, followed by sulfidation, resulted in a different surface and mesopore structure compared to the bare γ-Al_2_O_3_ support. However, it remains unclear whether these changes are solely due to the solids deposition and transformation, or whether the γ-Al_2_O_3_ support itself undergoes structural modification. To investigate this, MoS_2_ particles were segmented from the γ-Al_2_O_3_ matrix, allowing for a separate analysis of the support and the active phase. This segmentation, illustrated in Fig. 3(a–d), reveals distinct structural features.

The reliability of this analysis hinges on three key factors: the degree of sulfidation, the imaging accuracy, and the segmentation accuracy. Bulk surface analysis by XPS indicates sulfidation degrees of 73% for Mo/γ-Al_2_O_3_ and 74% for MoNiP/γ-Al_2_O_3_, while STEM-EDX indicates values of 79% and 89%, respectively. The large discrepancy observed for MoNiP/γ-Al_2_O_3_ likely stems from the presence of sulfided Ni species, which can be detected separately by XPS but are indistinguishable from sulfided Mo by STEM-EDX. Incomplete sulfidation results in residual oxidic Mo dispersed over the γ-Al_2_O_3_ surface, complicating the full isolation of the γ-Al_2_O_3_ support structure. Additionally, due to the planar, layered structure of MoS_2_ and the limitations imposed by the missing wedge in ET, only MoS_2_ slabs oriented parallel or slightly tilted (± several degrees) relative to the electron beam at each tilt angle are reliably reconstructed.^87,88^ Finally, visual inspection confirms that the segmentation process successfully isolates a representative portion of the MoS_2_ slabs, as shown in Fig. 3(b and d). Nonetheless, more sophisticated machine learning segmentation techniques could improve the accuracy of the segmentation, although these methods fall outside the scope of this study. Considering these aspects, we conclude that semi-quantitative analysis is feasible for both the isolated γ-Al_2_O_3_ support (discussed in the next section) and the MoS_2_ slabs (discussed thereafter), while acknowledging the limitations introduced by incomplete sulfidation and the missing wedge.

### Surface and mesopore structure of isolated γ-Al_2_O_3_ after sulfidation

MoS_2_ slabs were isolated from the γ-Al_2_O_3_ matrix in sulfided Mo and MoNiP samples, allowing for a direct comparison between the isolated γ-Al_2_O_3_ and the fully segmented solids, including bare γ-Al_2_O_3_, sulfided Mo/γ-Al_2_O_3_, and sulfided MoNiP/γ-Al_2_O_3._ The removal of MoS_2_ from the γ-Al_2_O_3_ surface leads to an increase in PV and SSA, a decrease in the strut width; however, no significant change in pore size (Fig. 3(e and f)). These observations suggest that enough MoS_2_ was isolated to enable assessment of structural changes in γ-Al_2_O_3_, induced by sulfidation of Mo or MoNiP.

Irrespective of whether Mo or MoNiP was present during sulfidation, the isolated γ-Al_2_O_3_ structures exhibited similar PV, SSA, PSD, and strut width. Additionally, compared to the bare γ-Al_2_O_3_, both systems showed an increase in tortuosity and surface corrugation, with the latter evidenced by a broadening of the shape index curve and increased curvedness (Fig. 3(g–i)). At the bulk scale, changes in the Al environment in the presence of Mo or MoNiP are observed using XPS. In Fig. S3.4(a), XPS spectra of the Al 2p peak exhibit peak broadening after calcination and sulfidation, suggesting changes in the electronic properties, surroundings, and/or metal–support interactions.

The positioning of MoS_2_ on the γ-Al_2_O_3_ surface can be inferred from the effect of slab removal on shape index and curvedness. Upon removal of MoS_2_, a broadening of the shape index and curvedness, reflecting increased surface corrugation, is observed relative to the intact Mo/γ-Al_2_O_3_ and MoNiP/γ-Al_2_O_3_ samples. This suggests that MoS_2_ slabs reside on sharply curved, dome- and cup-like surfaces of the underlying γ-Al_2_O_3_ surface, which is more corrugated than the bare γ-Al_2_O_3_ (Fig. 3(j)). Overall, despite differences in precursor chemistry, γ-Al_2_O_3_ undergoes similar structural changes upon sulfidation in the presence of either Mo or MoNiP, and the resulting MoS_2_ particles appear to form in comparable surface locations.

### Structural descriptors of isolated MoS_2_ slabs

**Fig. 4:**
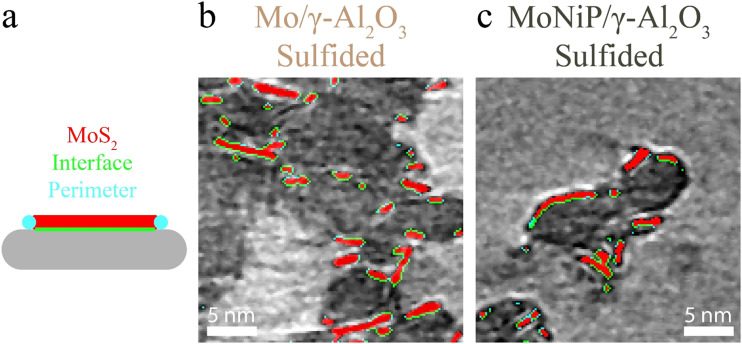
(a) Schematic of the MoS_2_ slab structure on a γ-Al_2_O_3_ surface. (b) and (c) show a zoom of a numerical slice (thickness = 0.19 nm) through cryo-ET reconstructions of sulfided Mo/γ-Al_2_O_3_ and MoNiP/γ-Al_2_O_3_, respectively. The MoS_2_ (red), interface (green), and perimeter (cyan) are annotated.

Direct quantification of MoS_2_ slabs revealed structural descriptors relevant to the understanding of catalyst functioning. To enable visual inspection of the MoS_2_ slabs, a schematic of the shape, as well as the interface with the support and perimeter, as shown in Fig. 4(a), is used to annotate the slabs in Fig. 4. Additionally, key descriptors obtained through XPS, STEM-EDX, and cryo-ET are summarized in Table 2.

**Table 2:** XPS, STEM-EDX, and cryo-ET quantification results of MoS_2_ slabs

		MoS_2_	MoS_2_NiP
Mo sulfidation degree (%)	XPS	73 ± 2	74 ± 5
	STEM-EDX	79 ± 6	89 ± 8
S loading (wt%)	XPS	6.5 ± 0.4	6.8 ± 0.3
	STEM-EDX	6.7 ± 0.4	6.6 ± 0.4
	Cryo-ET (in MoS_2_)^a^	5.8 ± 0.7	5.7 ± 1.6
Mo loading (wt%)	XPS	9.7 ± 0.4	9.5 ± 0.2
	STEM-EDX	12.0 ± 0.9	10.9 ± 0.5
	Cryo-ET (in MoS_2_)^a^	8.7 ± 1.0	8.6 ± 2.4
	Cryo-ET (MoS_2_ + MoO_*x*_)^b^	11.9 ± 1.4	11.6 ± 3.3
Structural descriptors found by cryo-ET	Average MoS_2_ thickness (nm)	0.51 ± 0.13	0.54 ± 0.05
	MoS_2_-γ-Al_2_O_3_ (m^2^ g^−1^ cat)	15.2 ± 3.6	16.1 ± 0.9
	MoS_2_-γ-Al_2_O_3_ (m^2^ g^−1^ MoS_2_)	170	178
	Total slab perimeter (m^2^ g^−1^ cat)	8.1 ± 0.8	6.6 ± 2.0
	Average NN distance (nm)	2.50 ± 0.05	2.70 ± 0.20

a Corrected to theoretical thickness and for missing wedge.

b Corrected to theoretical thickness, for missing wedge, and incomplete sulfidation.

The weight loading of Mo and S can be estimated based on the segmented MoS_2_. However, the following corrections need to be applied to get a realistic estimate. First, about 30% of the MoS_2_ slabs are not (properly) visualized due to the missing wedge (SI, section S6). Second, the distribution of MoS_2_ slab thickness needs to be corrected towards the theoretical thickness (0.65 nm). Third, the incomplete sulfidation needs to be accounted for when calculating the total Mo wt%. To this end, the sulfidation degree found through XPS was used. The cryo-ET-derived Mo weight loading present in MoS_2_ is 8.7 ± 1.0 wt% and 8.6 ± 2.4 wt% for Mo/γ-Al_2_O_3_ and MoNiP/γ-Al_2_O_3_, respectively. The total Mo weight loading (in MoS_2_ and MoO_*x*_) is 11.9 ± 1.4 wt% and 11.6 ± 3.3 wt% for Mo/γ-Al_2_O_3_ and MoNiP/γ-Al_2_O_3_, respectively, which aligns quite well with XPS and STEM-EDX measurements.

The interface between the MoS_2_ slabs and the γ-Al_2_O_3_ provides insight into slab orientation. Visual inspection, supported by quantitative analysis, reveals a mixture of basal- and edge-plane slab connections to the γ-Al_2_O_3_. The normalized MoS_2_-γ-Al_2_O_3_ interface areas are 170 m^2^ g^−1^ MoS_2_ for Mo/γ-Al_2_O_3_ and 178 m^2^ g^−1^ MoS_2_ for MoNiP/γ-Al_2_O_3_, compared to the theoretical maximum of 325 m^2^ g^−1^ MoS_2_ (SI, section S6). This effect is likely due to the thinner and more uniformly distributed oxidic Mo phase in the NiP-promoted catalyst, which, after sulfidation, results in a larger total MoS_2_ surface area in contact with the support. A lower interface area suggests a greater proportion of edge-plane connections. Additionally, the total slab perimeter reflects the interface accessible to reactants. The presence of Ni and P results in a smaller average perimeter, although the standard deviation overlaps with the Mo-only system. Finally, the average nearest-neighbor (NN) distance between slabs is comparable for Mo/γ-Al_2_O_3_ and MoNiP/γ-Al_2_O_3_.

Establishing correlations between structural descriptors obtained after catalyst preparation and activation, and catalytic performance is crucial in catalysis research. Although no catalytic tests were performed in this study, it is well established that MoNiP/γ-Al_2_O_3_ exhibits enhanced HDS activity compared to Mo/γ-Al_2_O_3_ due to the presence of Ni.^33,35,36,89,90^ Only small structural differences, accompanied by significant variability, are observed between the slabs in the Mo/γ-Al_2_O_3_ and MoNiP/γ-Al_2_O_3_ catalyst. This may suggest that cryo-ET lacks the resolution to capture the promotional effects, which are known to arise from the formation of Ni–Mo–S active sites, where Ni is preferentially located at the sulfur-terminated MoS_2_ edges.^90^ Moreover, the S-edge/M-edge ratio plays a critical role in catalytic performance and can be tuned through synthesis conditions and additives.^16^ Baubet *et al.* demonstrated through high-resolution HAADF-STEM that the 2D shape index of MoS_2_ slabs directly correlates with the exposure of M- and S-edges, and thus catalytic activity,^88^ highlighting opportunities for the 3D assessment performed in this study. However, achieving such correlations requires higher imaging resolution and more sophisticated segmentation, followed by quantitative 3D slab analysis (*e.g.*, shape index of individual slabs). Exploring these 3D quantitative structure–function relationships at the nanoscale remains largely unexplored for catalysts in general, providing a promising direction for future research.

## Conclusions

This study demonstrates the capability of cryo-ET to resolve the 3D surface and mesopore structure of bare γ-Al_2_O_3_ supports and γ-Al_2_O_3_-supported hydrotreating catalysts after calcination and subsequent sulfidation. While bare γ-Al_2_O_3_ largely retains its structural integrity during calcination and sulfidation, cryo-ET reveals subtle variations in surface and pore descriptors that remain inaccessible to bulk characterization techniques. Deposition of oxidic Mo or MoNiP induces modest restructuring of the intrinsic γ-Al_2_O_3_ structure. The Mo-only catalyst shows more pronounced surface corrugation and pore tortuosity than the MoNiP catalyst, consistent with thicker Mo deposition in the absence of Ni and P, in line with trends observed in bulk characterization and literature. Following sulfidation, both Mo/γ-Al_2_O_3_ and MoNiP/γ-Al_2_O_3_ display overall comparable structural characteristics. Visual assessment of the γ-Al_2_O_3_ and the segmentation of MoS_2_ slabs from its surface implies local restructuring of the underlying support. Moreover, slab segmentation indicates that they are preferentially located on sharply curved dome- and cup-like regions of the γ-Al_2_O_3_ surface. Despite the established influence of P and Ni on MoS_2_ morphology and HDS performance, no significant differences in the 3D structural descriptors of the sulfided catalyst were detected. These findings emphasize the need for higher-resolution 3D imaging, more advanced segmentation approaches, and deeper 3D structural analysis to enable robust structure–function correlations capable of establishing a fundamental framework for identifying optimum catalyst configurations beyond the specific catalysts investigated in this study.

## Author contributions

J. M. J. J. Heinrichs: conceptualization, data curation, formal analysis, investigation, methodology, project administration, resources, software, validation, visualization, writing – original draft. Th. Weber: conceptualization, funding acquisition, validation, supervision, writing – reviewing & editing. H. Friedrich: conceptualization, validation, supervision, writing – reviewing & editing. J. Zečević: conceptualization, validation, writing – reviewing & editing. A. Evtushkova: formal analysis, investigation, writing – reviewing & editing. E. J. M. Hensen: conceptualization, funding acquisition, validation, supervision, writing – reviewing & editing.

## Conflicts of interest

There are no conflicts to declare.

## Supplementary Material

CY-016-D5CY01396H-s001

## Data Availability

The data supporting this article have been included as part of the supplementary information (SI). Supplementary information (SI): S1. Calculation of corrugation value; S2. Untreated, calcined, and sulfided γ-Al_2_O_3_ – cross sections & segmentation of Fig. 1(a–c), and pore size distribution; S3. Bulk characterization of empty γ-Al_2_O_3_, Mo/γ-Al_2_O_3_, and MoNiP/γ-Al_2_O_3_; S4. Cryo-ET descriptor ranking of empty γ-Al_2_O_3_, Mo/γ-Al_2_O_3_, and MoNiP/γ-Al_2_O_3_; S5. Calcined & sulfided Mo(NiP)/γ-Al_2_O_3_ – zoom, cross sections & segmentation of Fig. 2(a–d) and S6. Theoretical surface area of MoS_2_ and missing wedge correction; S7. References. See DOI: https://doi.org/10.1039/d5cy01396h.
