# Supplementary material for: Cryogenic electron tomography reveals the mesoporous structure evolution during γ-Al2O3 supported Mo and MoNiP catalyst formation
Source: Catal Sci Technol. 2026 Mar 5;16(7):2331–44. doi: 10.1039/d5cy01396h (PMC12962062; doi:10.1039/d5cy01396h)
Supplement: CY-016-D5CY01396H-s001 [file CY-016-D5CY01396H-s001.pdf]

This journal is © The Royal Society of Chemistry 2026

Supplementary Information

Cryogenic Electron Tomography Reveals the Mesoporous Structure Evolution during γ-Al_2O_3 supported Mo and MoNiP Catalyst Formation

Jason M.J.J. Heinrichs[a,b], Angelina Evtushkova[a], Jovana Zečević[c], Thomas Weber[a,d], Heiner Friedrich[b,e], and Emiel J.M. Hensen[a,*]

[a)] *Laboratory of Inorganic Materials and Catalysis, Department of Chemical Engineering and Chemistry, Eindhoven University of Technology (TU/e), Den Dolech 2, 5600 MB Eindhoven, The Netherlands*

[b)] *Center for Multiscale Electron Microscopy, Department of Chemical Engineering and Chemistry, Eindhoven University of Technology (TU/e), Den Dolech 2, 5600 MB Eindhoven, The Netherlands*

[c)] *Shell Global Solutions International B.V., Grasweg 31, 1031 HW Amsterdam, The Netherlands*

[d)] *Retired from Shell Global Solutions International B.V., Grasweg 31, 1031 HW Amsterdam, The Netherlands*

[e)] *Laboratory of Physical Chemistry, Department of Chemical Engineering and Chemistry, Eindhoven University of Technology (TU/e), Den Dolech 2, 5600 MB Eindhoven, The Netherlands*

** Corresponding author: Emiel J.M. Hensen: e.j.m.hensen@tue.nl*

Contents

S1. Calculation of corrugation value 2
S2. Untreated, calcined, and sulfided γ-Al_2O_3 – cross sections, segmentation of Figure 1 (a – c), and pore size distribution 3
S3. Bulk characterization of empty γ-Al_2O_3, Mo/γ-Al_2O_3, and MoNiP γ-Al_2O_3 8
S4. Cryo-ET descriptor ranking of empty γ-Al_2O_3, Mo/γ-Al_2O_3, and MoNiP γ-Al_2O_3 19
S5. Calcined & sulfided Mo(NiP)/γ-Al_2O_3 – zoom, cross sections & segmentation of Figure 2 (a – d) 20
S6. Theoretical surface area of MoS_2 and missing wedge correction 26
S7. References 27

S1. Calculation of corrugation value

Surface characteristics inaccessible to bulk characterization are shape index (SI) and curvedness (CVD). The SI is a measure of the local surface type, whereas the CVD is a measure of the bending degree of the local surface type, as is illustrated in **Figure S2.1**. For each individual reconstructed particle, the SI and CVD are calculated at each triangular patch of the isosurface, forming a distribution of both. Together, these descriptors can be used to describe the total corrugation of a single reconstructed particle.

Here, the corrugation is defined as the fluctuation in different surface types. There are two relevant fluctuations: (1) the shape type variation; and (2) the strength of the curvature variation. A high corrugation is caused by a broad SI distribution spread (i.e., variance) and/or a broad CVD distribution spread (i.e., variance). The corrugation can be calculated based on equation 1, where both the SI and CVD are treated as orthogonal contributions to the corrugation (i.e., they do not influence or interact with each other). The variance of the shape index and curvedness can be calculated with equation 2. To have equal contributions of both fluctuation types, it is important to scale the SI and CVD to [0 1]. The average SI and CVD can be calculated with equation 3, where i is the bin centre of the distribution.

$$Equation\ 1 \qquad Corrugation = \sqrt{{\sigma_{SI}}^2 + {\sigma_{CVD}}^2}$$

$$Equation\ 2 \qquad Variance\ shape\ index: {\sigma_{SI}}^2 = \frac{\sum_i \left(n_{counts,i}\left(SI_i - \mu_{SI}\right)^2\right)}{\sum_i n_{counts,i}}$$

$$Equation\ 3 \qquad Average\ shape\ index: \mu_{SI} = \frac{\sum_i \left(n_{counts,i} \times SI_i\right)}{\sum_i \left(n_{counts,i}\right)}$$

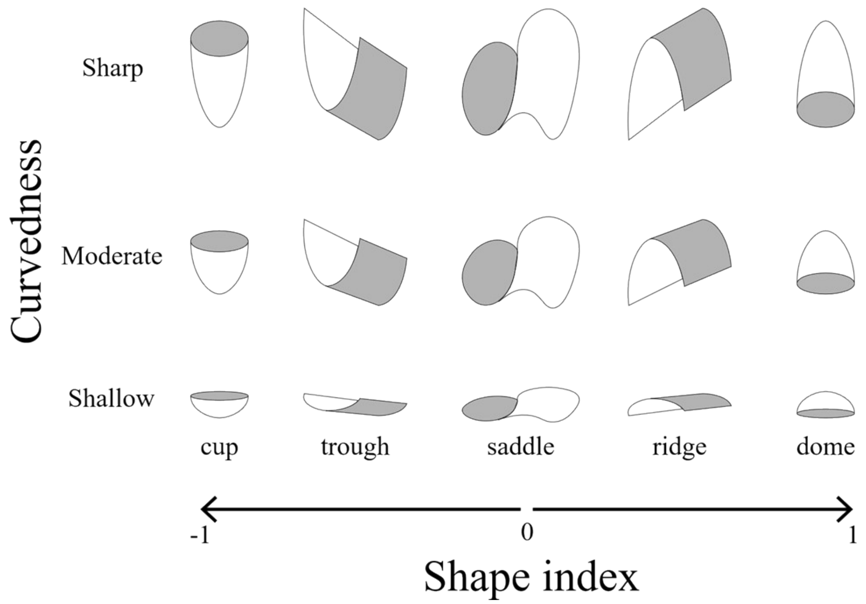

Figure S1.1 Terminology of the different local surface type (i.e., shape index) and bending degree of the local surface type (i.e., curvedness).

S2. Untreated, calcined, and sulfided γ-Al_2O_3 – cross sections, segmentation of Figure 1 (a – c), and pore size distribution

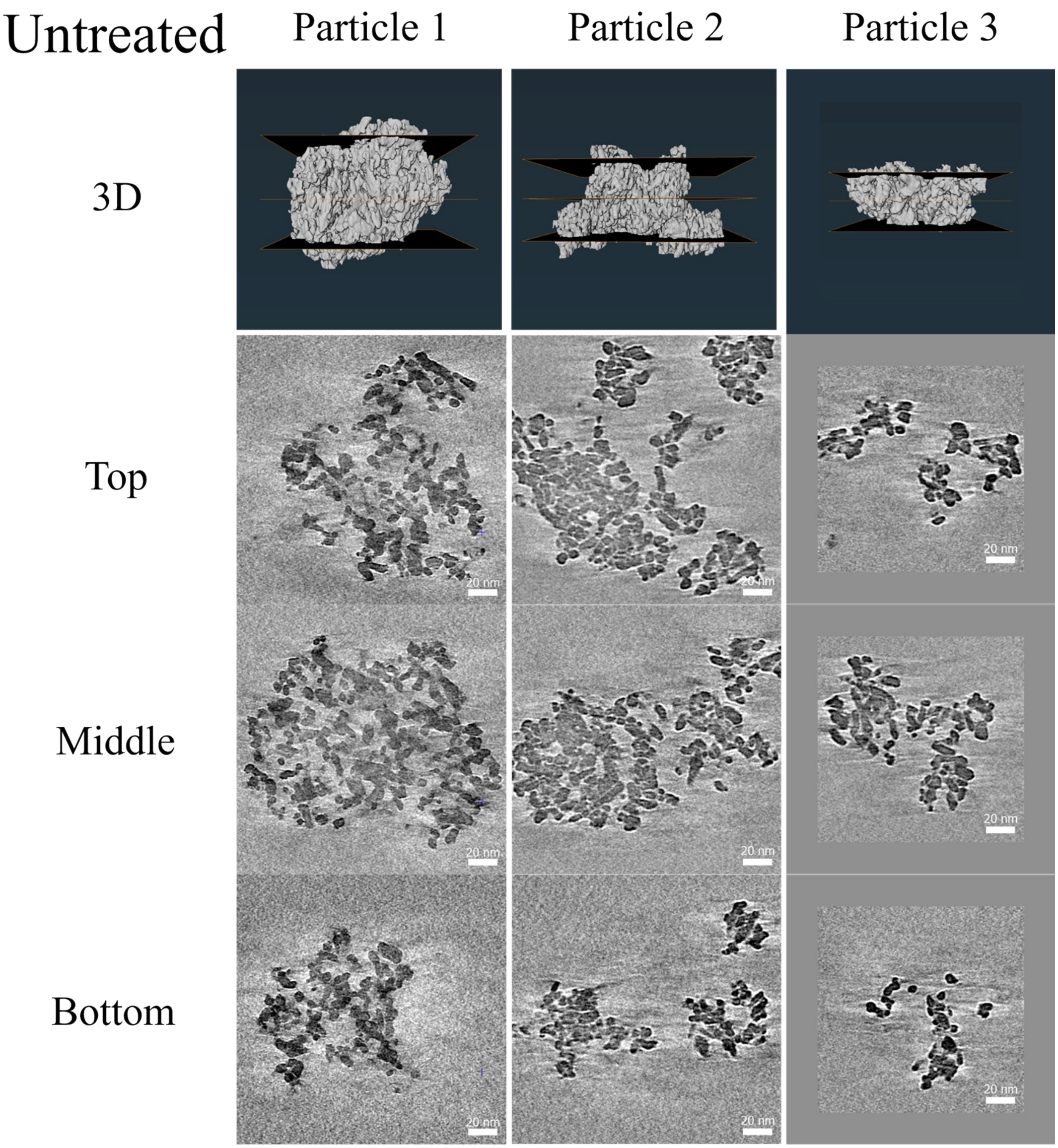

Figure S2.1 Numerical cross sections (thickness = 0.31 nm) through cryo-ET reconstructions of three untreated bare γ-Al_2O_3 particles used for qualitative and quantitative analysis. A 3D rendering of each particle is used to illustrate to height of the top, middle, and bottom numerical cross section.

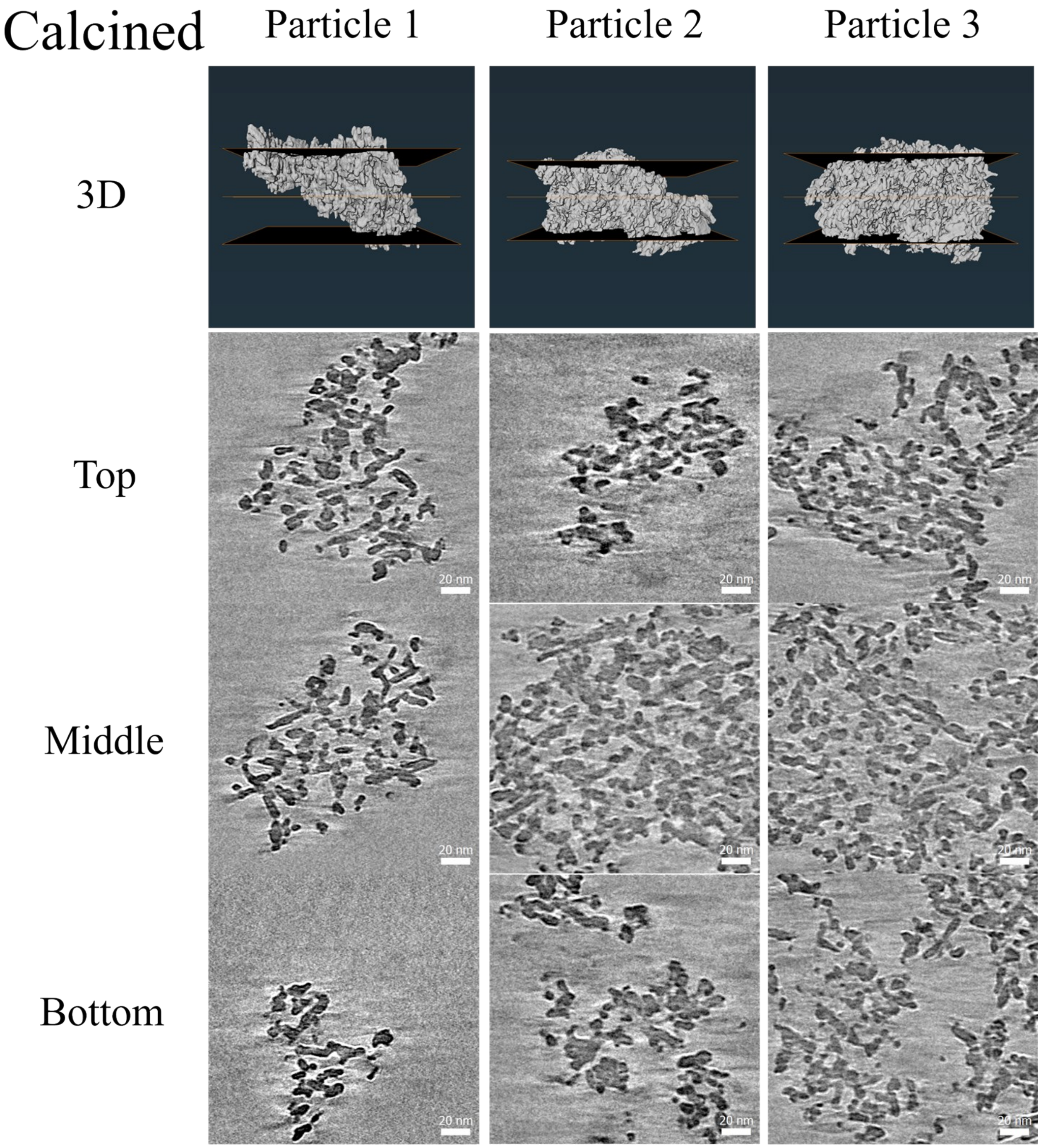

Figure S2.2 Numerical cross sections (thickness = 0.31 nm) through cryo-ET reconstructions of three calcined bare γ-Al_2O_3 particles used for qualitative and quantitative analysis. A 3D rendering of each particle is used to illustrate to height of the top, middle, and bottom numerical cross section.

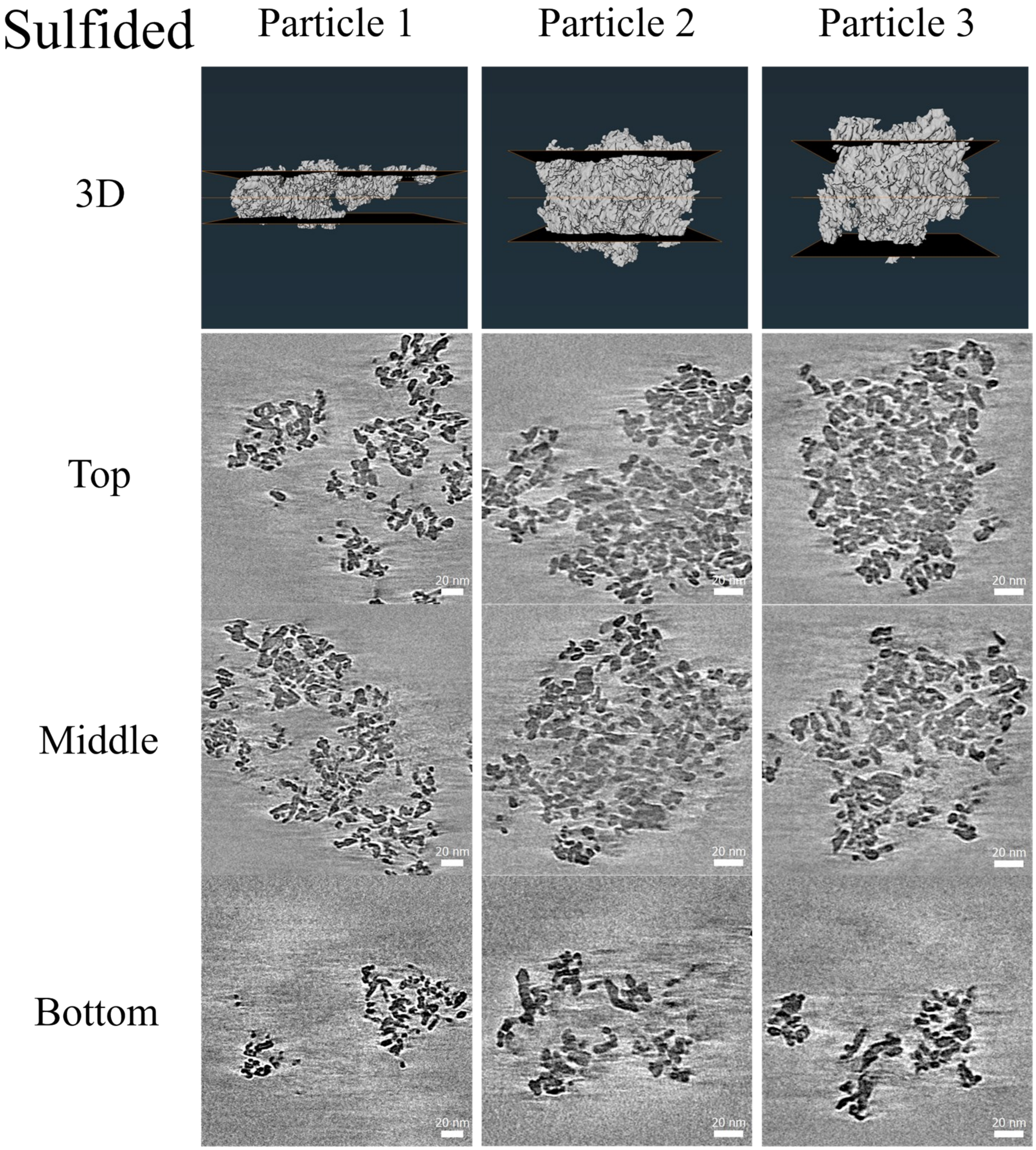

Figure S2.3 Numerical cross sections (thickness = 0.31 nm) through cryo-ET reconstructions of three sulfided bare γ-Al_2O_3 particles used for qualitative and quantitative analysis. A 3D rendering of each particle is used to illustrate to height of the top, middle, and bottom numerical cross section.

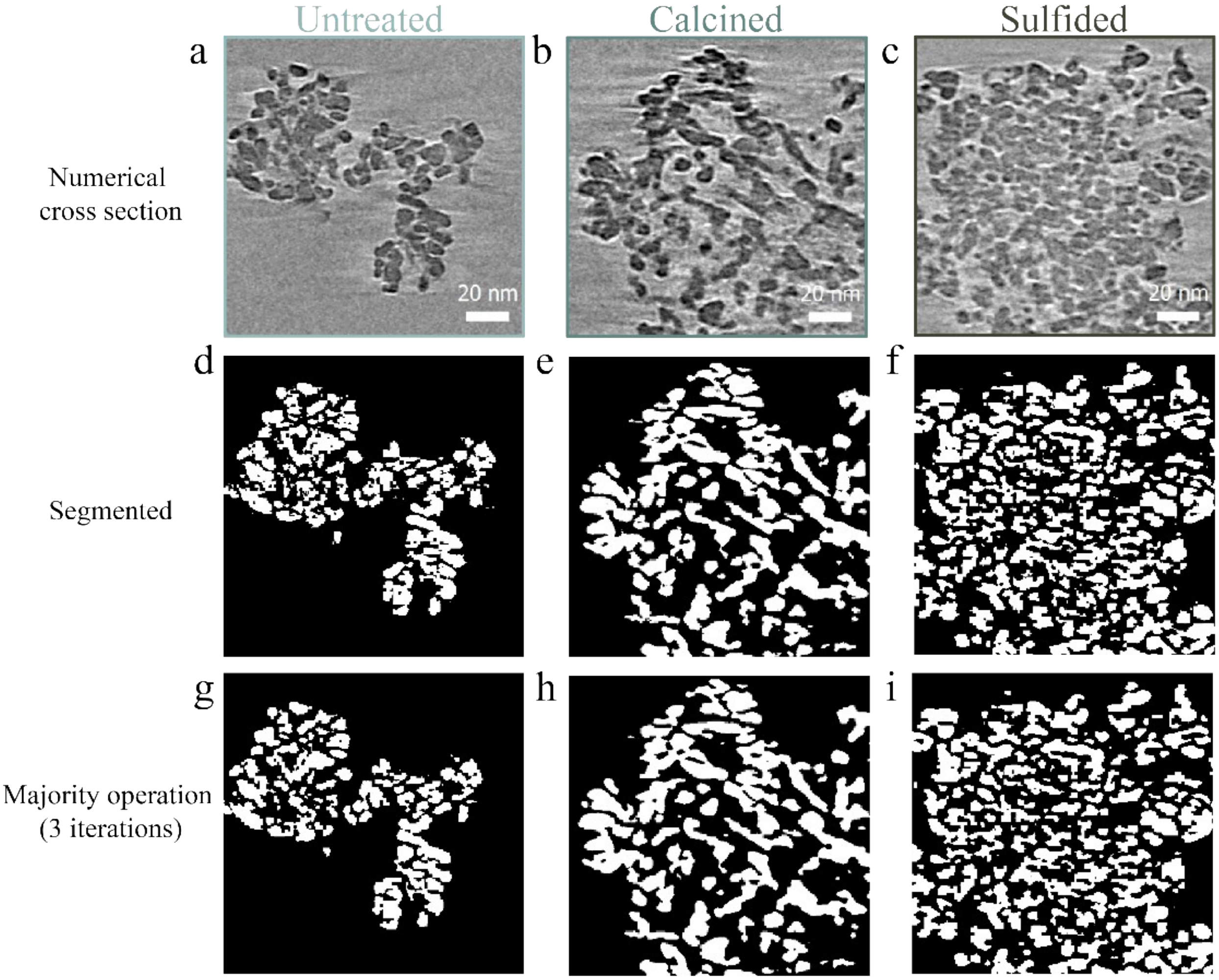

Figure S2.4 Example of the image processing performed on cryo-ET reconstructions of the bare γ-Al_2O_3 particles: (a – c) show the median filtered (kernel size: 3x3x3) numerical cross sections through cryo-ET reconstructions (thickness = 0.31 nm) of bare, calcined, and sulfided bare γ-Al_2O_3 particles; (d – f) and (g – i) show the segmented slices, corresponding to the numerical slices shown in (a – c), before and after 3 majority iterations, respectively.

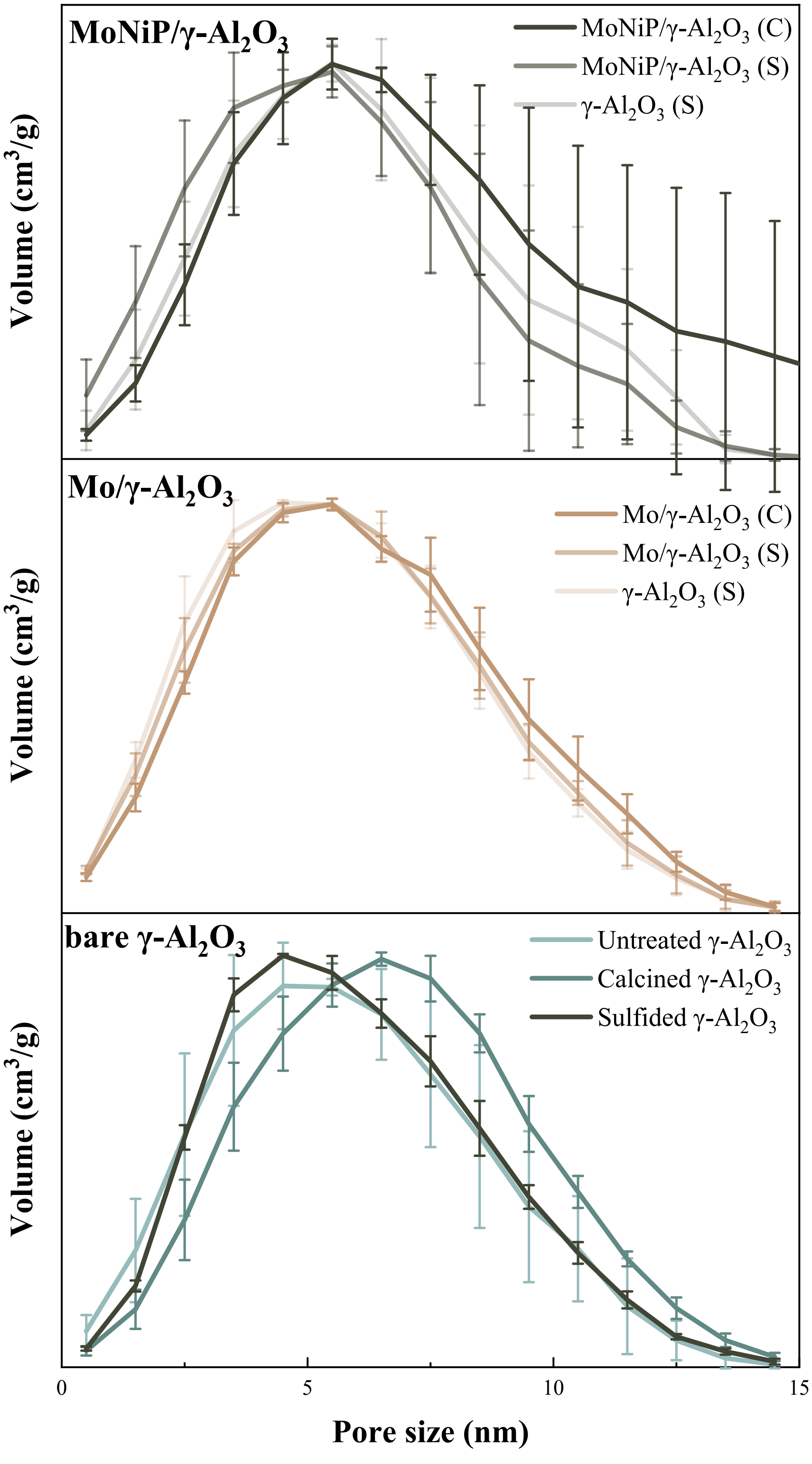

Figure S2.5. Pore size distributions of the 3D reconstructed volumes obtained via cryo-ET. Bottom: bare γ-Al_2O_3 support in its untreated, calcined, and sulfided state. Middle: calcined and sulfided Mo/γ-Al_2O_3 and the isolated γ-Al_2O_3 in its sulfided state. Top: calcined and sulfided MoNiP/γ-Al_2O_3 and the isolated γ-Al_2O_3 in its sulfided state.

S3. Bulk characterization of empty γ-Al_2O_3, Mo/γ-Al_2O_3, and MoNiP γ-Al_2O_3

N_2 physisorption

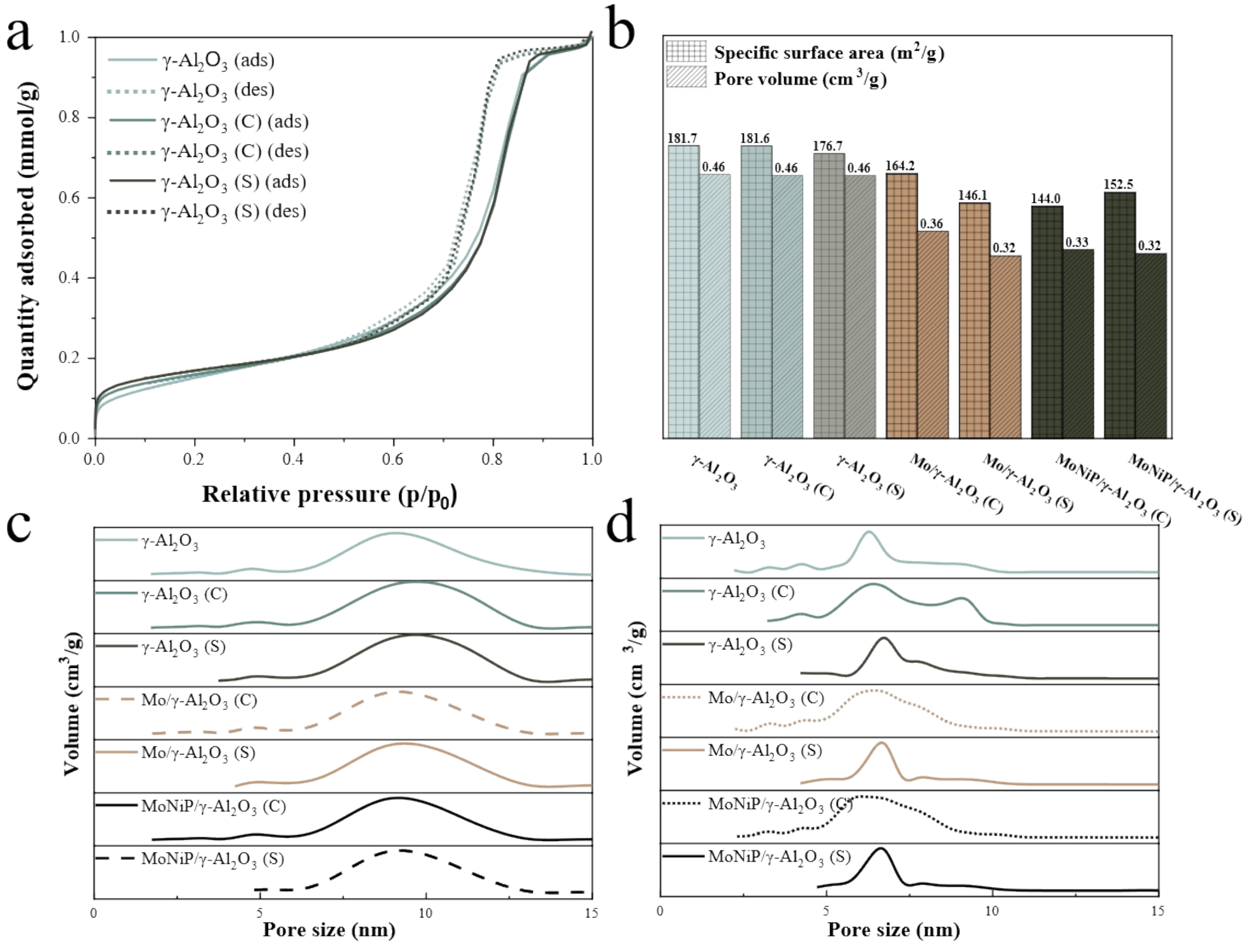

Figure S3.1 N_2 physisorption results of bare γ-Al_2O_3, Mo/γ-Al_2O_3, and MoNiP/γ-Al_2O_3 after calcination and sulfidation: (a) adsorption and desorption isotherm of untreated (γ-Al_2O_3), calcined (γ-Al_2O_3 (C)), and sulfided (γ-Al_2O_3 (S)) bare γ-Al_2O_3; (b) specific surface area (m^2/g) and pore volume (cm^3/g) obtained for each system; (c) the adsorption BJH PSD and (d) the desorption BJH PSD shown as the volumetric contribution (cm^3/g) per pore width range.

Table S3.1 PV and SSA obtained through cryo-ET and N_2 physisorption. The change in percentage, relative to the bare γ-Al_2O_3 is calculated for both techniques.

	PV (cm^3/g)		SSA (m^2/g)	
	**Cryo-ET**	**N_2 physisorption**	**Cryo-ET**	**N_2 physisorption**
γ-Al_2O_3	0.28 (ref)	0.46 (ref)	176 (ref)	180 (ref)
Mo/γ-Al_2O_3 - calcined	0.22 (−21 %)	0.36 (−22 %)	163 (−7 %)	164 (− 9%)
Mo/γ-Al_2O_3 - sulfided	0.17 (−39 %)	0.32 (−30%)	153 (− 13 %)	146 (− 19%)
MoNiP/γ-Al_2O_3 - calcined	0.21 (−25 %)	0.33 (−28 %)	145 (− 18 %)	144 (− 20 %)
MoNiP/γ-Al_2O_3 - sulfided	0.16 (−43 %)	0.32 (− 30%)	160 (− 9 %)	153 (− 15%)

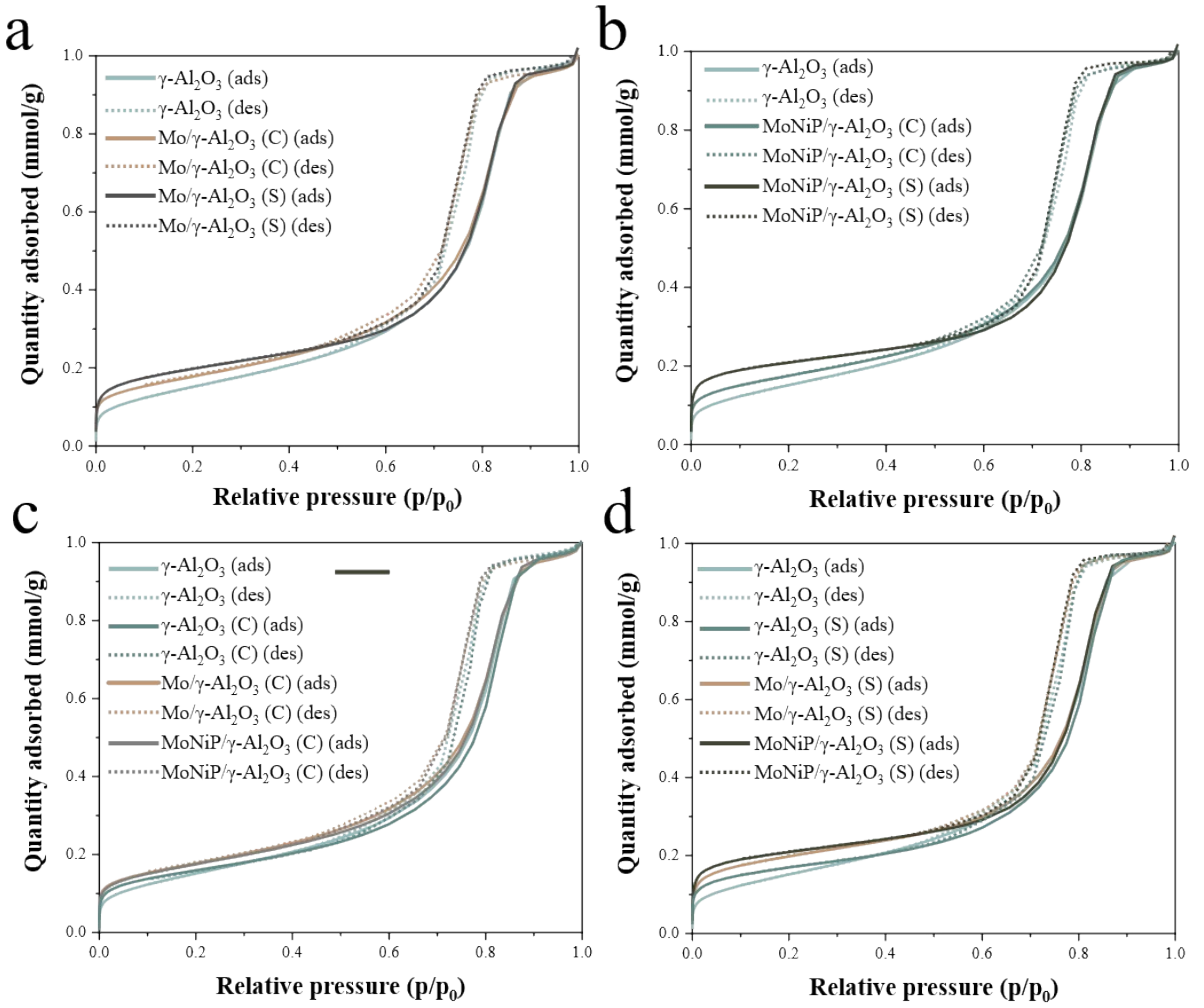

Figure S3.2 N_2 physisorption adsorption and desorption isotherms of bare γ-Al_2O_3, Mo/γ-Al_2O_3, and MoNiP/γ-Al_2O_3 after calcination and sulfidation: (a) untreated bare γ-Al_2O_3, calcined Mo/γ-Al_2O_3, and sulfided Mo/γ-Al_2O_3; (b) untreated bare γ-Al_2O_3, calcined MoNiP/γ-Al_2O_3, and sulfided MoNiP/γ-Al_2O_3; (c) untreated bare γ-Al_2O_3, calcined bare γ-Al2O3, calcined Mo/γ-Al_2O_3, and calcined MoNiP/γ-Al_2O_3; (d) untreated bare γ-Al_2O_3, sulfided bare γ-Al2O3, sulfided Mo/γ-Al_2O_3, and sulfided MoNiP/γ-Al_2O_3;

Synchrotron X-Ray Diffraction (XRD)

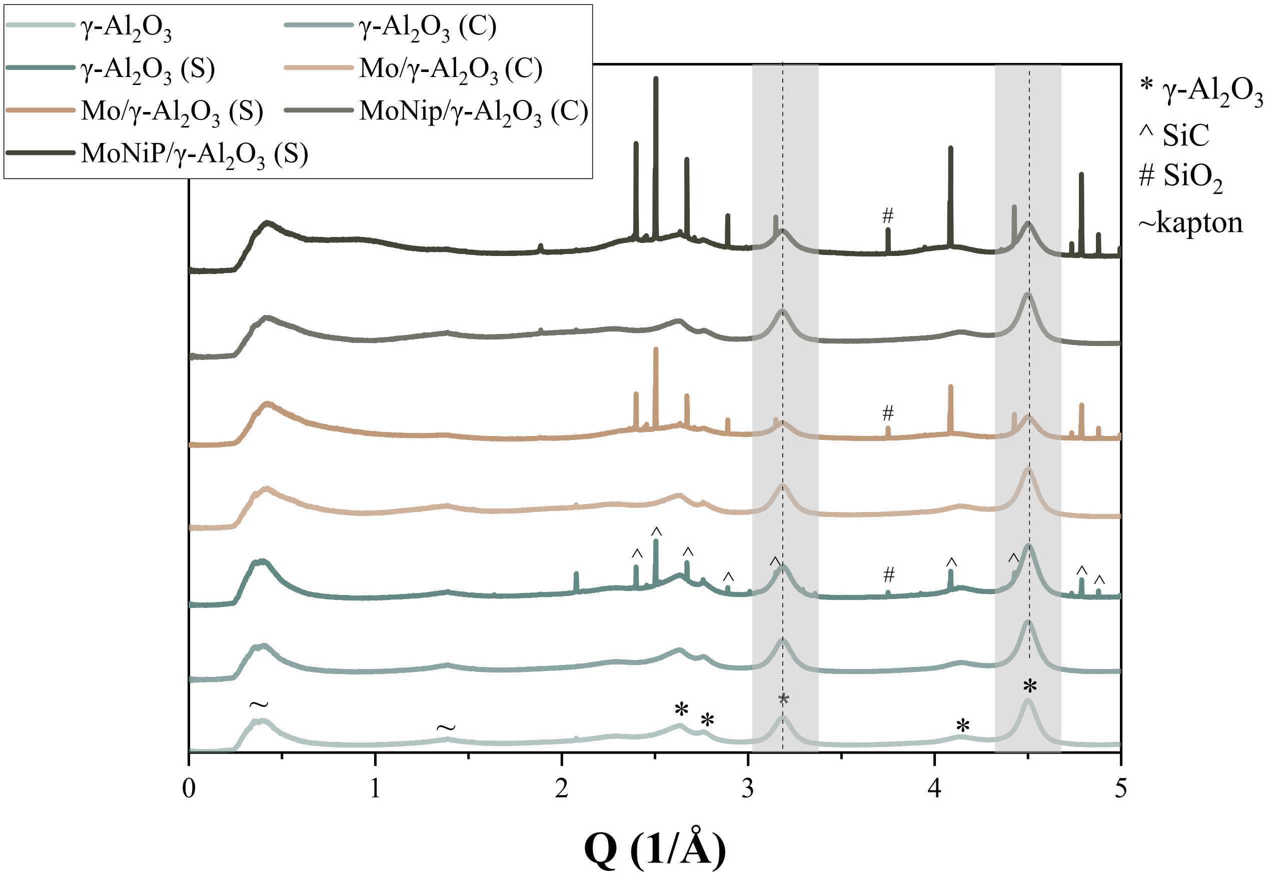

Figure S3.3 Synchrotron XRD patterns.

Table S3.2. Scherrer equation. Peak fitting was done in Origin.

Q (1/Å) (*hkl*)	**γ-Al_2O_3**	**γ-Al_2O_3 (C)**	**γ-Al_2O_3 (S)**	**Mo/γ-Al_2O_3 (C)**	**Mo/γ-Al_2O_3 (S)**	**MoNiP/γ-Al_2O_3 (C)**	**MoNiP/γ-Al_2O_3 (S)**
	Crystallite size (nm)						
2.6 (311)	4.2	4.2	4.0	4.1	5.2	5.2	5.4
2.8 (222)	8.4	8.4	8.4	7.9	8.9	9.5	9.4
3.2 (400)	5.4	5.3	5.2	5.4	5.4	5.4	5.5
4.1 (511)	4.4	4.6	4.3	4.3	3.9	4.3	2.7
4.5 (440)	5.9	5.7	5.9	5.9	5.8	5.7	5.5
Average	5.7	5.6	5.6	5.5	5.8	5.8	5.7
Std	1.5	1.5	1.6	1.4	1.7	1.9	2.1

X-ray Photoelectron Spectroscopy (XPS)

By means of the software CasaXPS, a standard procedure involving calibration towards the Al 2p signal (74.1 eV) and Shirley background subtraction was performed on all spectral data. Fitting was performed with a symmetric pseudo-Voigt function (GL(30)). Al 2p spectra were deconvoluted into $2p_{3/2}$ and $2p_{1/2}$ doublets and were used for the determination of the total Al 2p area. O 1s spectra were deconvoluted into three peaks to measure the total area. These peaks possibly originate from (a combination of) Al_2O_3, MoO_3, NiO, and P_2O_5. However, further specification was out-of-scope. Mo 3d spectra were deconvoluted into Mo^{4+} (as in MoS_2), Mo^{5+} (as in MoS_xO_y), and Mo^{6+} (as in MoO_3) doublets with splitting energy of 3.15 eV.[1–5] Ni $2p_{3/2}$ spectra were deconvoluted into Ni^{2+} with a satellite peak at around + 6 eV (as in NiO, NiS, or NiMoS).[2,5] NiS and NiMoS could not be distinguished and were deconvoluted as one. P 2p spectra were deconvoluted into $2p_{3/2}$ and $2p_{1/2}$ doublets. S 2p spectra were deconvoluted into two doublets, terminal disulfide and/or sulfide (S^{2-}) ligands and bridging disulfide ligands (S_2^{2-}). [3–5]

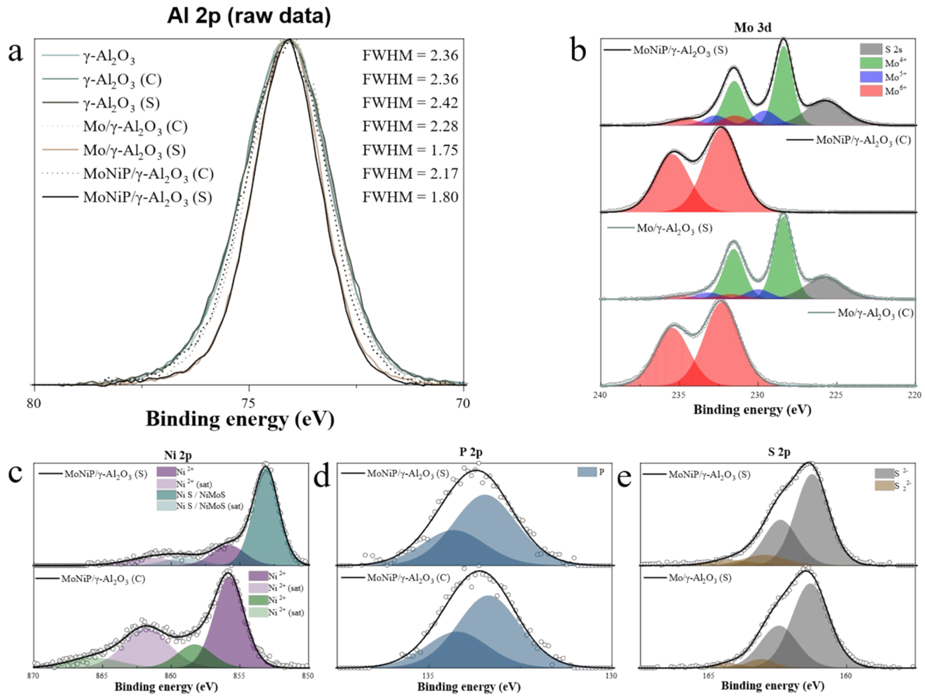

Figure S3.4 XPS results after calcination and sulfidation: (a) raw spectra of the Al 2p in bare γ-Al_2O_3, Mo/γ-Al_2O_3, and MoNiP/γ-Al_2O_3; (b) fitting of Mo 3d spectra of Mo/γ-Al_2O_3 and MoNiP/γ-Al_2O_3; fitting of Ni 2p spectra (c), P 2p spectra (d), and S 2p spectra (d) of MoNiP/γ-Al_2O_3.

XPS spectra in **Figure S3.4 (b)** revealed that, after deposition and calcination, only oxidic Mo^{VI} species, as in MoO_3, are present in both the Mo/γ-Al_2O_3 and MoNiP/γ-Al_2O_3. After sulfidation, XPS (**Figure S3.4**) reveals a mixture of Mo^{IV} species as in MoS_2, Mo^{V} species as in intermediate oxysulfidic Mo, and Mo^{VI} species as in residual MoO_3 are detected, with sulfidation degrees of approximately 73% for Mo/γ-Al_2O_3 and 74% for MoNiP/γ-Al_2O_3.[2,6] No differences in sulfur speciation are observed between the two catalysts.

For MoNiP/γ-Al_2O_3, Ni exists predominantly as NiO_x after deposition and calcination, and as NiS_x, $NiMo_xS_x$, and NiO_x species after sulfidation. The speciation of P remains unchanged throughout the process.

The degree of sulfidation, at% and wt% were calculated by the following equation:

$$Degree\ of\ sulfidation = \frac{[Mo^{4+}]}{[Mo^{4+}] + [Mo^{5+}] + [Mo^{6+}]}$$

$$at\%_X = \frac{\frac{[X]}{SF_X}}{\sum_i \frac{[X_i]}{SF_i}} \times 100\%,$$

where SF = Sensitivity factor according to CasaXPS library for K-Alpha instrument (Al 2p = 0.5371; O 1s = 2.93; Mo 3d = 9.5; Ni 2p = 14.61; P 2p = 1.192; S 2p = 1.677)

$$wt\%_X = \frac{at\%_X \cdot M_X}{\sum_i at\%_i \cdot M_i}$$

, where M is the molar mass of X

Table S3.3 Peak locations found through XPS. Each sample was measured in triplo for wt% quantification.

	S1_cp_10Mo	S1_sp_10Mo	S1_cp_10MoNiP	S1_sp_10MoNiP
	B.E. (eV)	*B.E. (eV)*	*B.E. (eV)*	*B.E. (eV)*
Mo^{4+} $3d_{5/2}$	-	228.44 ± 0.04	-	228.50 ± 0.09
Mo^{4+} $3d_{3/2}$	-	231.59 ± 0.04	-	231.65 ± 0.09
Mo^{5+} $3d_{5/2}$	-	229.93 ± 0.05	-	230.60 ± 0.62
Mo^{5+} $3d_{3/2}$	-	233.08 ± 0.05	-	233.72 ± 0.62
Mo^{6+} $3d_{5/2}$	232.39 ± 0.06	231.85 ± 0.09	232.29 ± 0.04	231.24 ± 1.18
Mo^{6+} $3d_{3/2}$	235.54 ± 0.06	235.00 ± 0.09	235.54 ± 0.04	234.39 ± 1.18
S 2s	-	225.87 ± 0.05	-	225.84 ± 0.05
NiO_x	-	-	855.87 ± 0.05	853.27 ± 0.12
NiO_x *(sat)*	-	-	861.89 ± 0.17	860.28 ± 0.84
NiO_x	-	-	857.98 ± 0.48	-
NiO_x *(sat)*	-	-	864.31 ± 1.65	-
NiS / NiMoS	-	-	-	855.73 ± 0.12
NiS / NiMoS (sat)	-	-	-	863.50 ± 1.41
$P\ 2p_{3/2}$	-	-	133.42 ± 0.04	133.43 ± 0.02
$P\ 2p_{1/2}$	-	-	134.28 ± 0.04	134.29 ± 0.02
S^{2-} $2p_{3/2}$	-	161.35 ± 0.04	-	161.36 ± 0.08
S^{2-} $2p_{1/2}$	-	162.50 ± 0.04	-	162.51 ± 0.08
S_2^{2-} $2p_{3/2}$	-	163.14 ± 0.11	-	163.06 ± 0.08
S_2^{2-} $2p_{1/2}$	-	164.29 ± 0.11	-	164.21 ± 0.08
Al 2p[1]	74.10 ± 0.00	74.10 ± 0.00	74.10 ± 0.00	74.10 ± 0.00
O 1s[2]	530.76 ± 0.04	530.84 ± 0.03	530.87 ± 0.04	530.88 ± 0.04

[1] fit of Al 2p by doublet for total area; [2] fit of O 1s by three peaks for total area.

Ultraviolet-Visible Diffuse Reflectance Spectroscopy (UV-VIS DRS)

UV-VIS DRS spectra of Mo/γ-Al_2O_3 and MoNiP/γ-Al_2O_3 are shown in **Figure S3.5**. The adsorption bands between 200 and 400 nm correspond to the ligand-to-metal charge transfer O^{2-} → Mo^{6+}.[9] In line with RAMAN, UV VIS DRS shows a highly dispersed mixture of isolated tetrahedral (band around 230 to 260 nm) molybdate species and octahedral (band around 280 to 320 nm) coordinated polymolybdate species.[10] The slight absorption increases in the range of 550 to 800 nm, which indicates the presence of octahedral Ni oxide species. Finally, the larger band-gap energy (E_g) for MoNiP/γ-Al_2O_3 suggests smaller particles compared to Mo/γ-Al_2O_3.

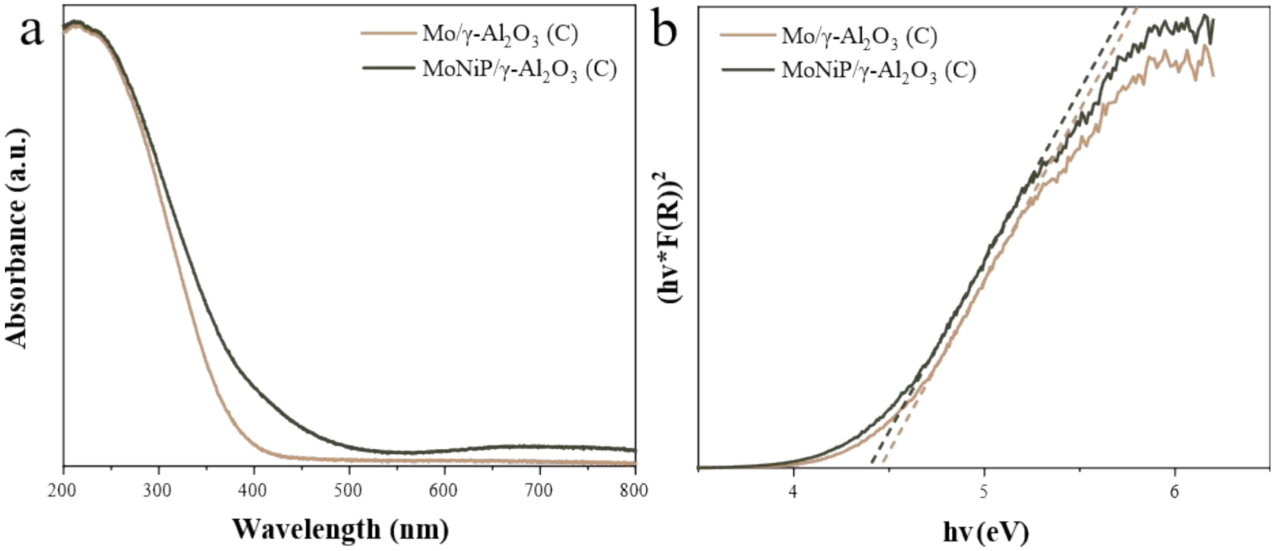

Figure S3.5 UV VIS DRS results of Mo/γ-Al_2O_3 and MoNiP/γ-Al_2O_3. (a)

Table S3.4 The electronic edge energy (E_g) of Mo/γ-Al_2O_3 and MoNiP/γ-Al_2O_3 found at the intercept between the x-axis and the tangent line of $[F(R_\infty hv]^2$ versus hv.

	**E_g (eV)**
Mo/γ-Al_2O_3	4.40
MoNiP/γ-Al_2O_3	4.45

Raman spectroscopy

Raman spectra of Mo/γ-Al_2O_3 and MoNiP/γ-Al_2O_3 (**Figure S3.6**) revealed adsorption peaks at 955 cm^{-1}, 910 cm^{-1}, 840 cm^{-1}, 566 cm^{-1}, 355 cm^{-1}, and 220 cm^{-1}. The bands at around 955 cm^{-1}, 910 cm^{-1}, 355 cm^{-1}, and 220 cm^{-1} are characteristic of vibrations within octahedrally coordinated polymolybdate species.[7,8] The broad band at around 840 cm^{-1} is characteristic of vibrations within isolated tetrahedrally coordinated molybdate. The band at 566 cm^{-1} is attributed to the Al-O stretching mode.[8] Both samples consist of a mixture of octahedral and tetrahedral coordinate molybdate species. However, the intensity differences observed in the bands at 840 cm^{-1} and 910 cm^{-1} suggest there are more tetrahedrally coordinated molybdate species in Mo/γ-Al_2O_3, exhibiting a stronger interaction with γ-Al_2O_3.[8]

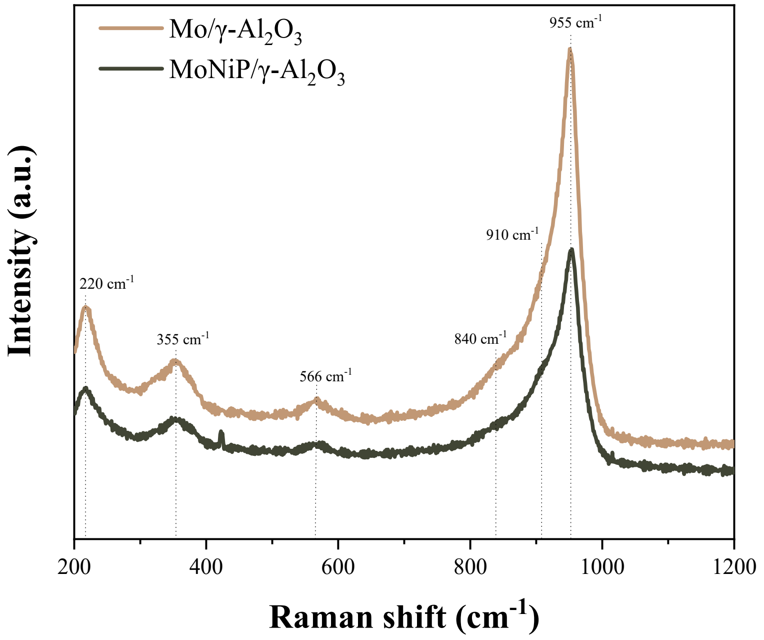

Figure S3.6 RAMAN spectra of Mo/γ-Al_2O_3 and MoNiP/γ-Al_2O_3.

H_2 – temperature programmed reduction (TPR)

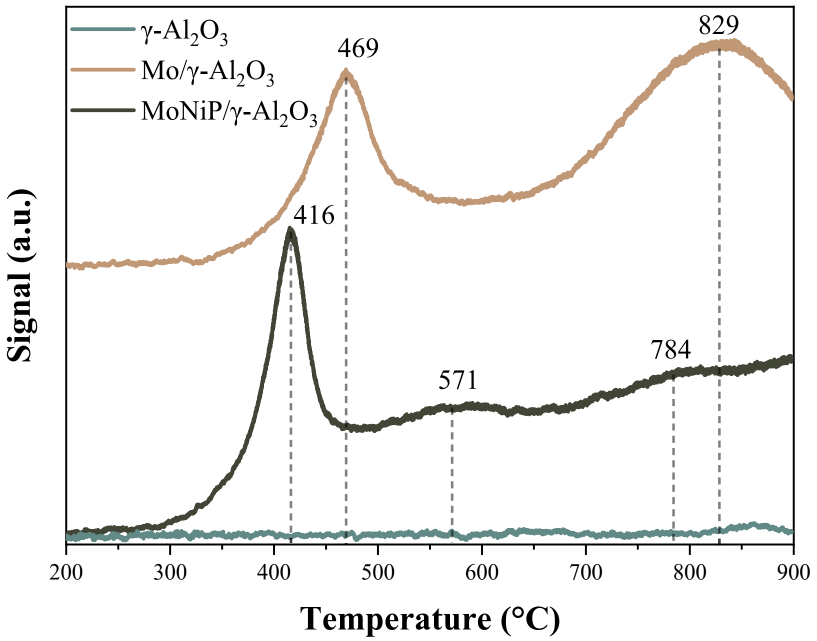

Figure S3.7 TPR of bare γ-Al_2O_3, Mo/γ-Al_2O_3 and MoNiP/γ-Al_2O_3.

Weight loading and atom percentage by ICP-OES, XPS and STEM-EDX

Table S3.5 Weight loadings according to ICP-OES, XPS and STEM-EDX.

Weight loading (wt%)	**Mo (10 wt%[1])**			**Ni (2.4 wt%[1])**			**P (1.3 wt%[1])**			**S**		
	ICP-OES	XPS	STEM-EDX[2]	ICP-OES	XPS	STEM-EDX[2]	ICP-OES	XPS	STEM-EDX[2]	ICP-OES	XPS	STEM-EDX[2]
**Calcined Mo/γ-Al_2O_3**	11.08 ± 0.00	12.68 ± 0.45	12.02 ± 1.37	-	-	-	-	-	-	-	-	-
**Sulfided Mo/γ-Al_2O_3**	-	9.68 ± 0.37	12.00 ± 0.85	-	-	-	-	-	-	-	6.45 ± 0.37	6.65 ± 0.39
**Calcined MoNiP/γ-Al_2O_3**	10.96 ± 0.04	11.97 ± 0.27	10.34 ± 0.82	2.51 ± 0.02	2.19 ± 0.26	2.05 ± 0.19	1.65 ± 0.02	1.61 ± 0.13	1.17 ± 0.26	-	-	-
**Sulfided MoNiP/γ-Al_2O_3**	-	9.53 ± 0.20	10.93 ± 0.50	-	2.49 ± 0.07	2.08 ± 0.11	-	1.67 ± 0.18	1.29 ± 0.17	-	6.78 ± 0.28	6.59 ± 0.44

[1] intended weight loading; [2] STEM-EDX is averaged over 5 individual particles

Table S3.6 Atom percent according to ICP-OES, XPS and STEM-EDX

Atom percent (%)	**Mo (2.2 at%[1])**			**Ni (0.9 at%[1])**			**P (0.9 at%[1])**			**S**		
	ICP-OES	XPS	STEM-EDX[2]	ICP-OES	XPS	STEM-EDX[2]	ICP-OES	XPS	STEM-EDX[2]	ICP-OES	XPS	STEM-EDX[2]
**Calcined Mo/γ-Al_2O_3**		2.97 ± 0.11	2.87 ± 0.40	-	-	-	-	-	-	-	-	-
**Sulfided Mo/γ-Al_2O_3**	-	2.29 ± 0.07	2.99 ± 0.26	-	-	-	-	-	-	-	4.53 ± 0.37	4.96 ± 0.37
**Calcined MoNiP/γ-Al_2O_3**		2.82 ± 0.08	2.43 ± 0.23		0.85 ± 0.10	0.79 ± 0.07		1.18 ± 0.10	0.86 ± 0.20	-	-	-
**Sulfided MoNiP/γ-Al_2O_3**	-	2.30 ± 0.07	2.66 ± 0.16	-	0.98 ± 0.03	0.83 ± 0.05	-	1.24 ± 0.14	0.98 ± 0.14	-	4.89 ± 0.24	4.89 ± 0.24

[1] intended atom percent; [2] STEM-EDX is averaged over 5 individual particles

STEM-EDX mapping

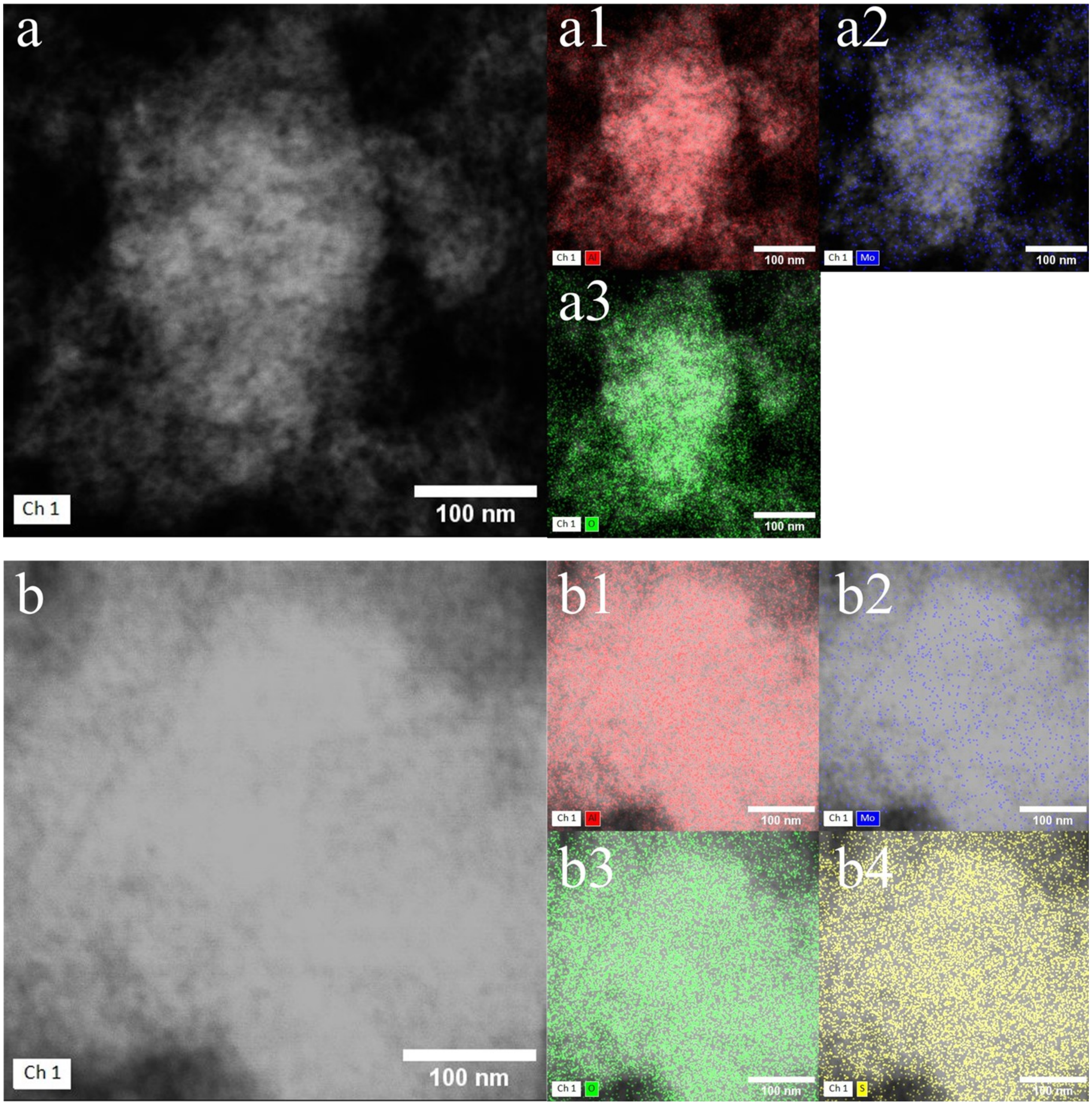

Figure S3.8 STEM-EDX mapping of calcined (a) and sulfided (b) Mo/γ-Al_2O_3.

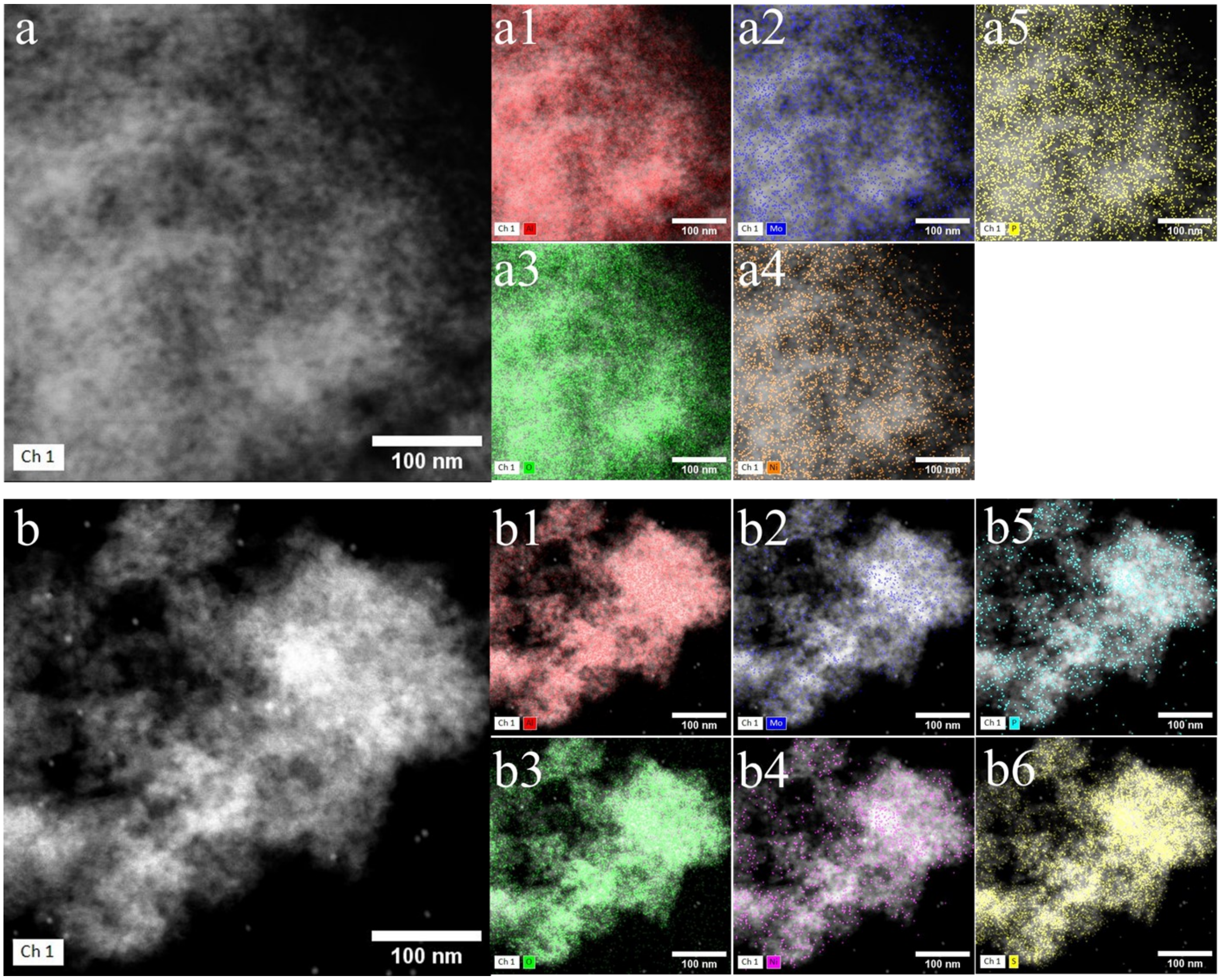

Figure S3.9 STEM-EDX mapping of calcined (a) and sulfided (b) MoNiP/γ-Al_2O_3.

S4. Cryo-ET descriptor ranking of empty γ-Al_2O_3, Mo/γ-Al_2O_3, and MoNiP γ-Al_2O_3

The combined set of descriptors obtained for each particle can be used to construct a structural model of the pore architecture that illustrates the relation among descriptors. This requires an overview of the descriptors for each state of bare γ-Al_2O_3 (**Table S3.1),** including a ranking from highest to lowest value for each descriptor. The structural model of the untreated γ-Al_2O_3 is as follows. It has the broadest distribution of shape index and curvedness, which implies a highly corrugated surface. This surface corrugation originates from its lowest average strut width, corresponding to the smallest primary particles. At last, a high surface corrugation leads to a high tortuosity.

Table S4.1 Ranking of the descriptors. (1) highest / broadest; (2) middle; (3) smallest / narrowest

	**Untreated**	**Calcined**	**Sulfided**
Shape index	1	3	2
Curvedness	1	3	2
SSA	2	3	1
Average struct width	3	1	2
Tortuosity	1	3	2
Pore volume	3	2	1
Average pore size	3	1	2

S5. Calcined & sulfided Mo(NiP)/γ-Al_2O_3 – zoom, cross sections & segmentation of Figure 2 (a – d)

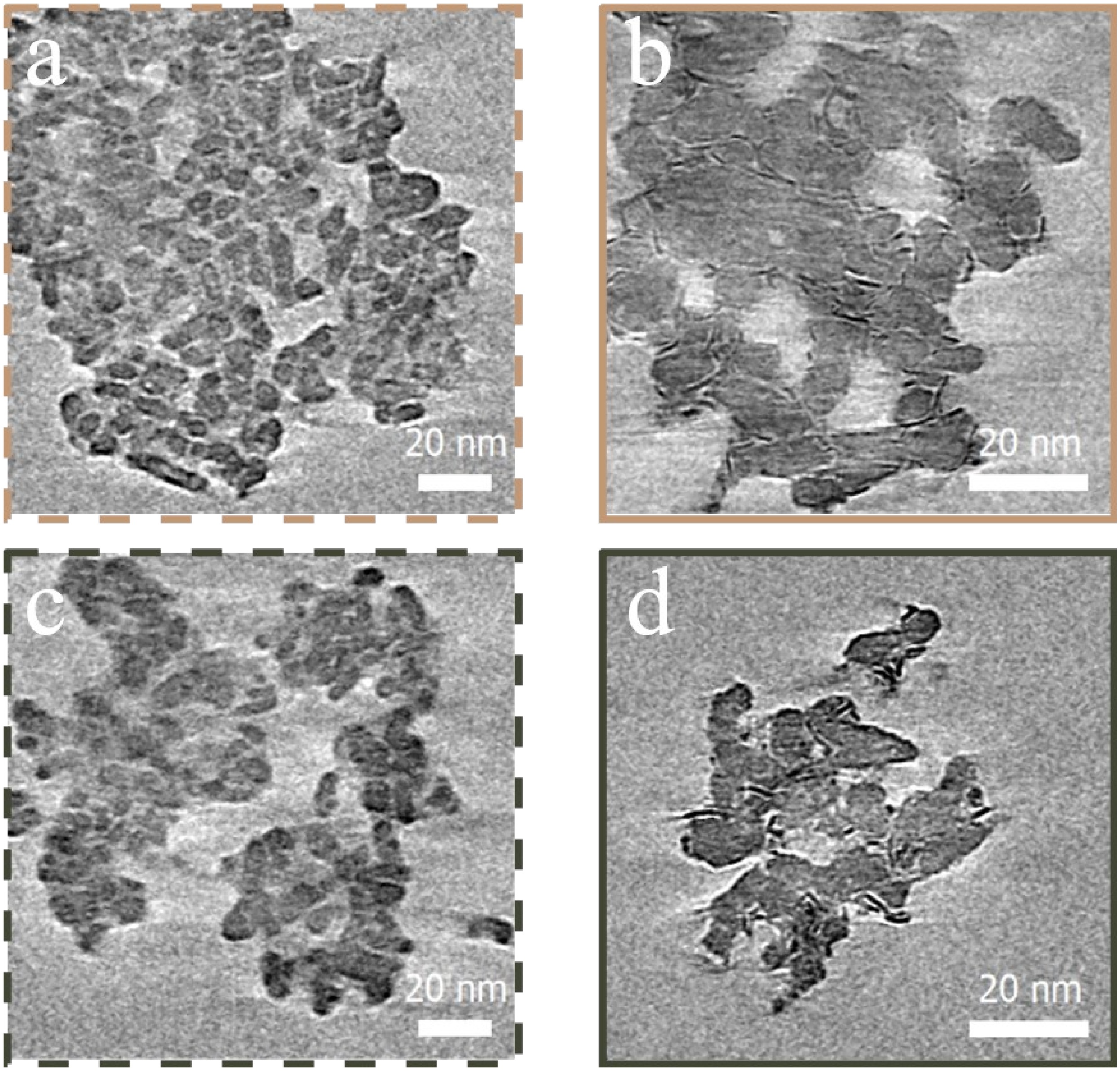

Figure S5.1 Numerical cross sections ((a and c) thickness = 0.31 nm; (b and d) thickness = 0.19 nm)) through cryo-ET reconstruction of calcined (a and c) and sulfided (b and d) Mo/γ-Al_2O_3 and MoNiP/γ-Al_2O_3. Enlarged view of **Figure 2** in the main text.

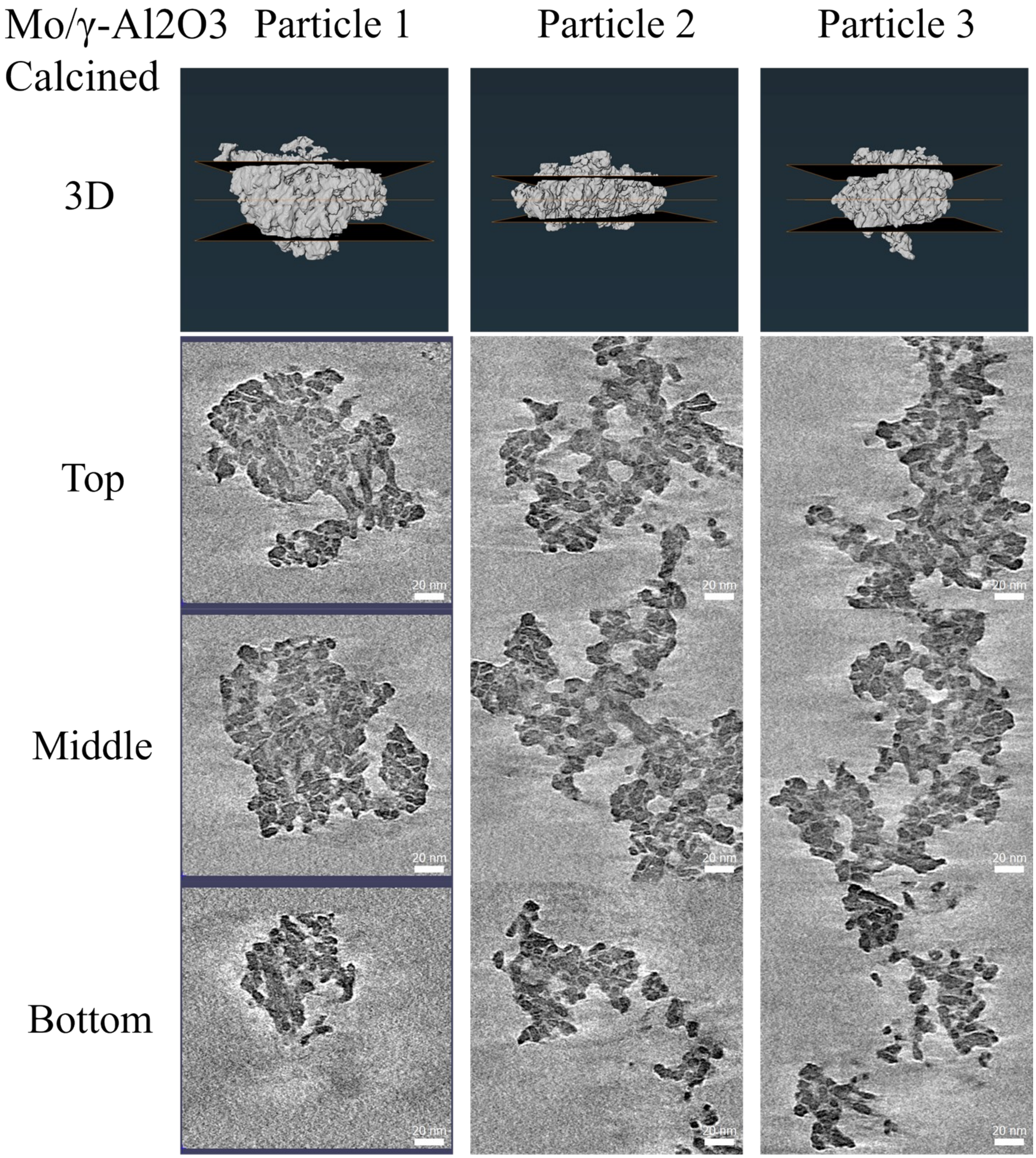

Figure S5.2 Numerical cross sections (thickness = 0.31 nm) through cryo-ET reconstructions of three calcined Mo/γ-Al_2O_3 particles used for qualitative and quantitative analysis. A 3D rendering of each particle is used to illustrate to height of the top, middle, and bottom numerical cross section.

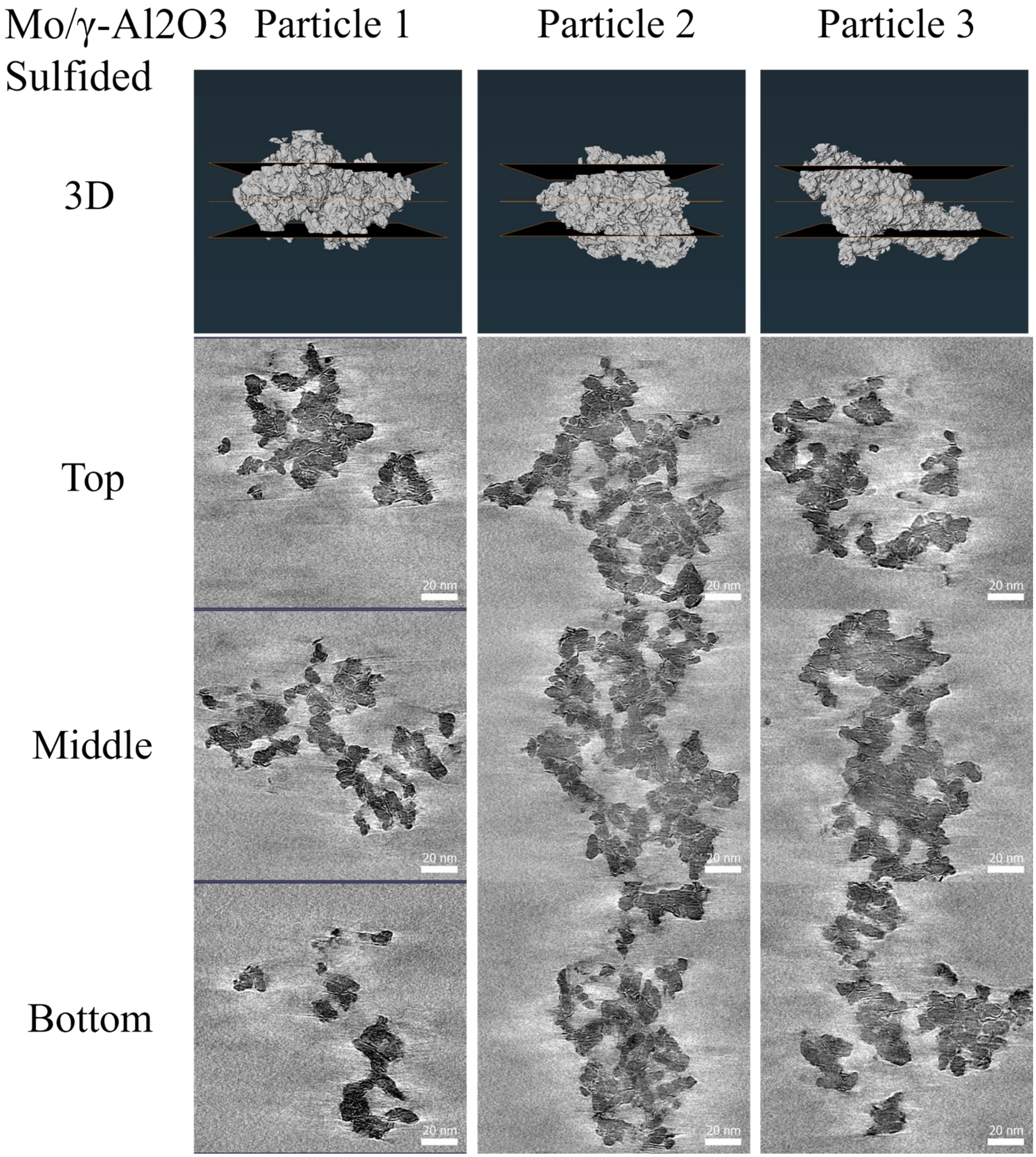

Figure S5.3 Numerical cross sections (thickness = 0.19 nm) through cryo-ET reconstructions of three sulfided Mo/γ-Al_2O_3 particles used for qualitative and quantitative analysis. A 3D rendering of each particle is used to illustrate to height of the top, middle, and bottom numerical cross section.

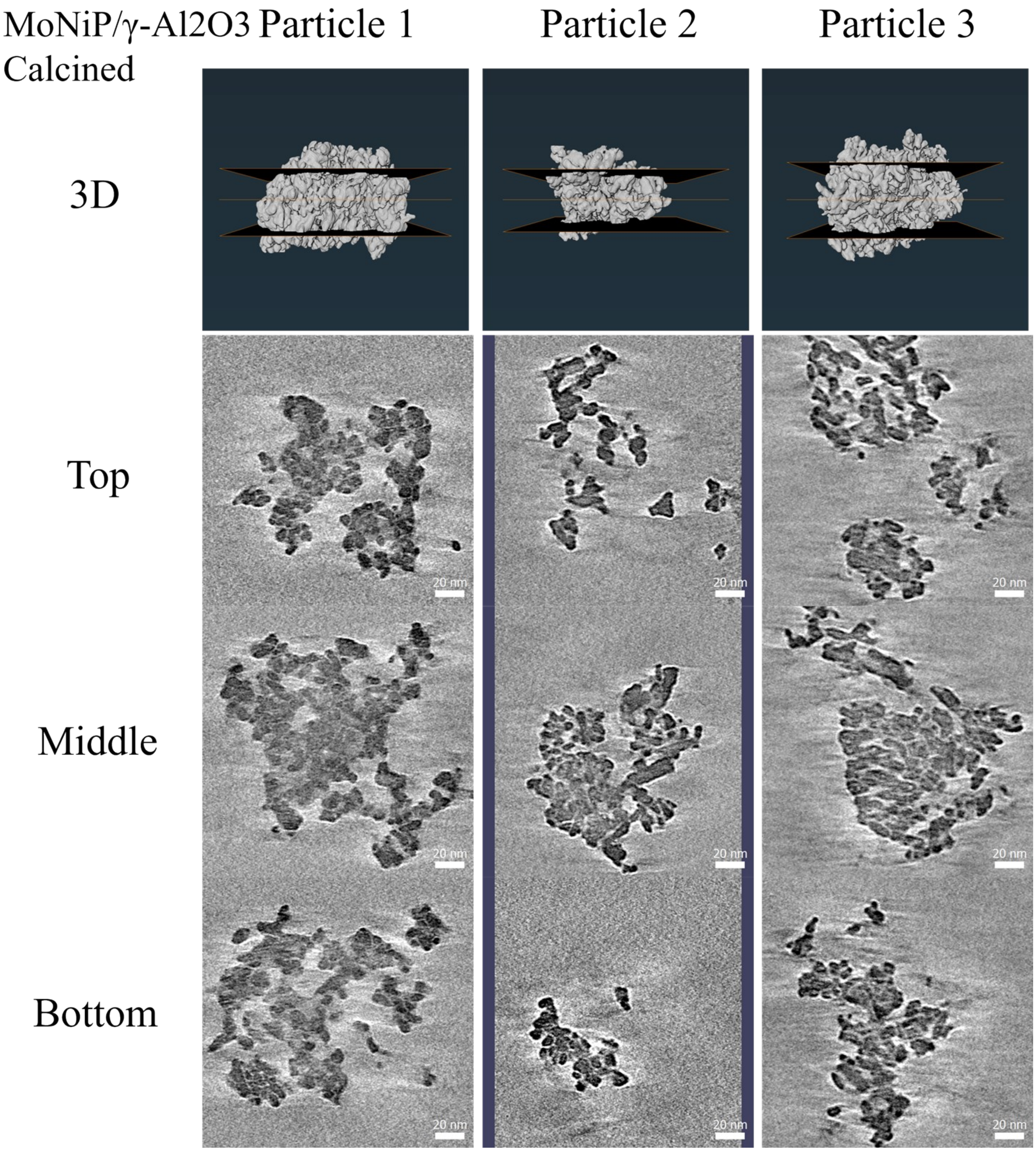

Figure S5.4 Numerical cross sections (thickness = 0.31 nm) through cryo-ET reconstructions of three calcined MoNiP/γ-Al_2O_3 particles used for qualitative and quantitative analysis. A 3D rendering of each particle is used to illustrate to height of the top, middle, and bottom numerical cross section.

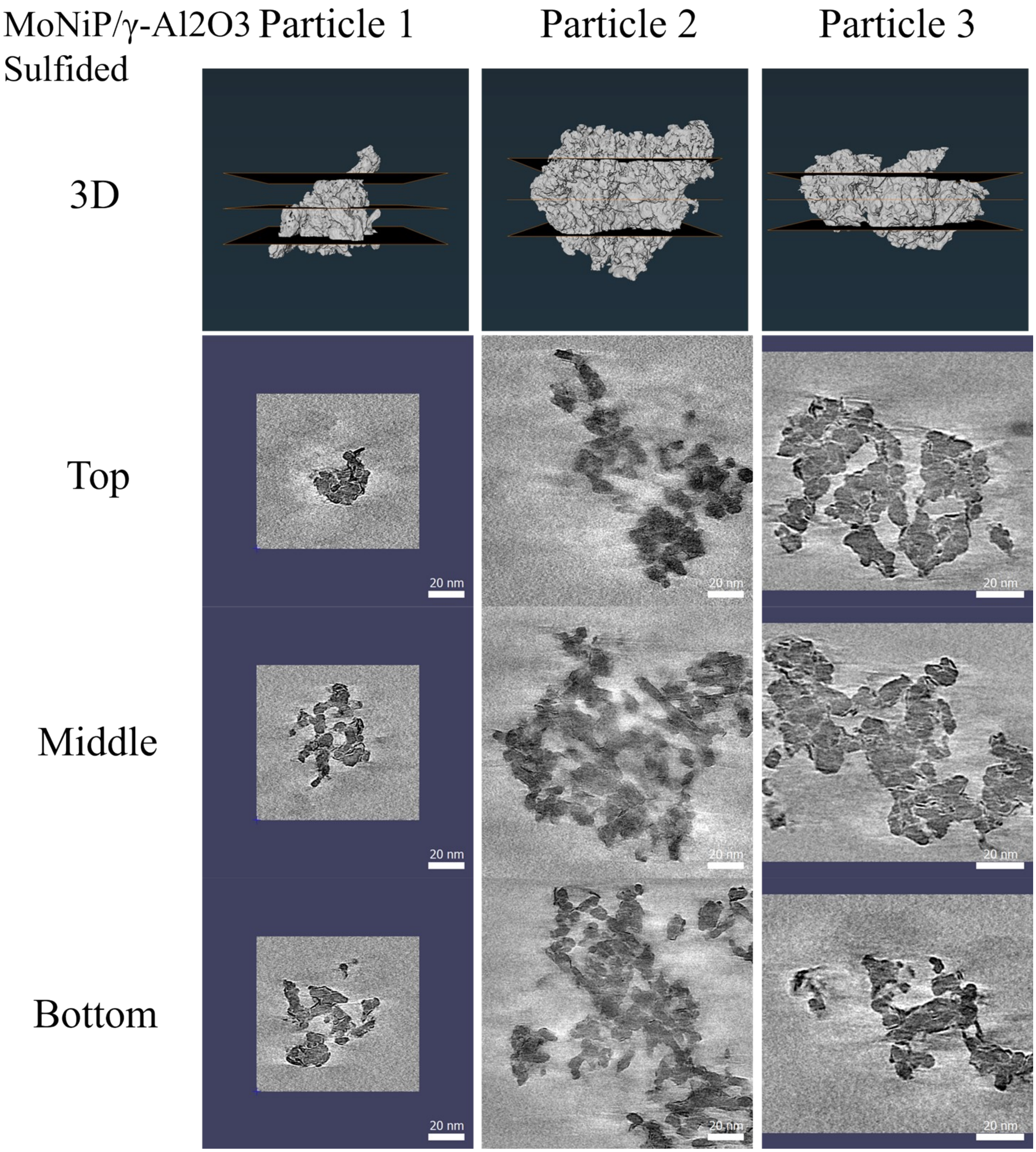

Figure S5.5 Numerical cross sections (thickness = 0.19 nm) through cryo-ET reconstructions of three sulfided MoNiP/γ-Al_2O_3 particles used for qualitative and quantitative analysis. A 3D rendering of each particle is used to illustrate to height of the top, middle, and bottom numerical cross section.

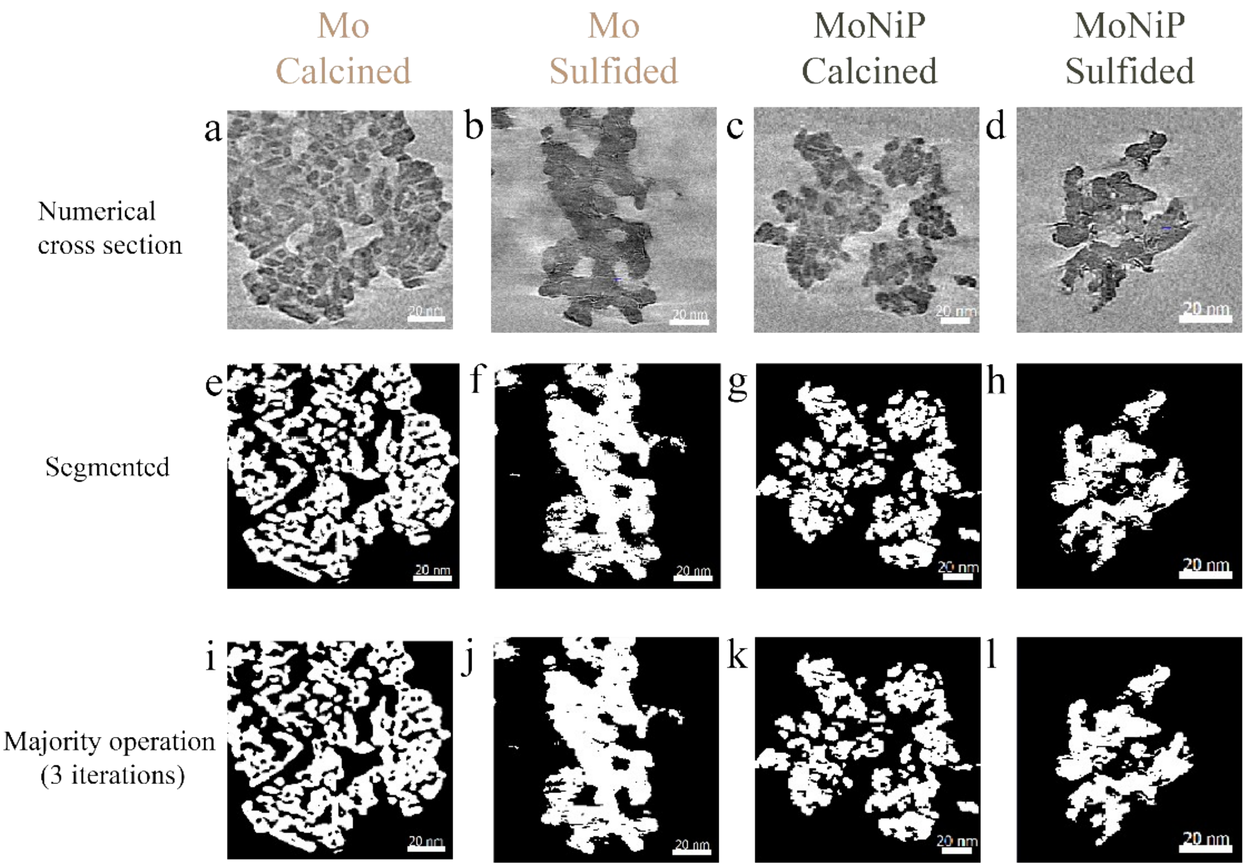

Figure S5.6 Example of the image processing performed on cryo-ET reconstructions of the calcined and sulfided Mo/γ-Al_2O_3 and MoNiP/γ-Al_2O_3 particles: (a and c) show the median filtered (kernel size: 3x3x3) numerical cross sections through cryo-ET reconstructions (thickness = 0.31 nm) of calcined Mo/γ-Al_2O_3 and MoNiP/γ-Al_2O_3; (b and d) show the median filtered (kernel size: 3x3x3) numerical cross sections through cryo-ET reconstructions (thickness = 0.19 nm) of sulfided Mo/γ-Al_2O_3 and MoNiP/γ-Al2O3 particles; (e – h) and (i – l) show the segmented slices, corresponding to the numerical slices shown in (a – d), before and after 3 majority iterations, respectively.

S6. Theoretical surface area of MoS_2 and missing wedge correction

Calculation of theoretical maximum MoS_2-γ-Al_2O_3 interface

Assumption: each layer consists out of one layer of Mo sandwiched between two layers of S. The value calculated represents the ideal case where every atom in a hexagonal structure contributes to the surface area and there is perfect exfoliation into monolayers without any stacking or aggregation. The in-plane lattice constant, a, is equal to 3.16 Å.

$$Unit\ cell\ area\ for\ primitive\ cell\ \text{in}\ hexagonal\ structures = \frac{\sqrt{3}}{2}a^2 = \frac{\sqrt{3}}{2}3.16^2 = 8.65\ \text{Å}^2 = 8.65\ \times 10^{-20}\ m^2$$

$$Number\ of\ MoS_2 units\ per\ gram = \frac{N_A}{mass\ Mo + 2 \times mass\ S} = \frac{6.022 \times 10^{23}}{95.95 + 2 \times 32.06} = 3.76 \times 10^{21} units/g$$

$$Theoretical\ MoS_2 - \gamma - Al_2O_3\ interface = 3.76 \times 10^{21} \times 8.65\ \times 10^{-20} \approx 325\ m^2/g\ MoS_2$$

Missing wedge correction

The missing wedge poses in problem in ET as it leads to an anisotropic resolution in the form of elongation and blurring of the objects in the z-direction.[11,12] As a consequence, it can be expected that not all MoS_2 slabs will be sufficiently visible for segmentation. Rather complex approaches to suppress the missing information and increase the resolution in the z-direction exist but are out of scope in this study. [11,12] However, a simple estimate, assuming isotropic orientation of the slabs in the disordered γ-Al_2O_3 matrix, is used to account for the loss in MoS_2.

The tilt range is ± 68 which gives a missing wedge of 180° - 136° = 44°. Hence the fraction of Fourier space which is not sampled is 43°/180° = 0.24. This means that 24% of the slabs are in orientations within the missing wedge. The correction factor is then equal to 1 / (1 - 0.24) = 1.31. This would indicate that there are about 31% more slabs present than imaged. This value will be used in the final section to correct the weight loading found.

S7. References

1 B. Baubet, E. Devers, A. Hugon, E. Leclerc and P. Afanasiev, *Appl. Catal. A Gen.*, 2014, **487**, 72–81.

2 L. Van Haandel, M. Bremmer, P. J. Kooyman, J. A. R. Van Veen, Th. Weber and E. J. M. Hensen, *ACS Catal.*, 2015, **5**, 7276–7287.

3 Th. Weber, J. C. Muijsers, J. H. M. C. Van Wolput, C. P. J. Verhagen and J. W. Niemantsverdriet, *J. Phys. Chem.*, 1996, **100**, 14144–14150.

4 Th. Weber, J. C. Muijsers and J. W. Niemantsverdriet, *J. Phys. Chem.*, 1995, **99**, 9194–9200.

5 G. M. Bremmer, L. Van Haandel, E. J. M. Hensen, J. W. M. Frenken and P. J. Kooyman, *J. Phys. Chem. C*, 2016, **120**, 19204–19211.

6 J. C. Muijsers, Th. Weber, R. M. Vanhardeveld, H. W. Zandbergen and J. W. Niemantsverdriet, *J. Catal.*, 1995, **157**, 698–705.

7 C. C. Williams, J. G. Ekerdt, J. M. Jehng, F. D. Hardcastle and I. E. Wachs, *J. Phys. Chem.*, 1991, **95**, 8791–8797.

8 Z. Liu, W. Han, D. Hu, S. Sun, A. Hu, Z. Wang, Y. Jia, X. Zhao and Q. Yang, *J. Catal.*, 2020, **387**, 62–72.

9 J. Xu, T. Huang and Y. Fan, *Appl. Catal. B Environ.*, 2017, **203**, 839–850.

10 O. Y. Gutiérrez and T. Klimova, *J. Catal.*, 2011, **281**, 50–62.

11 L. Paavolainen, E. Acar, U. Tuna, S. Peltonen, T. Moriya, P. Soonsawad, V. Marjomäki, R. H. Cheng and U. Ruotsalainen, *PLoS One*, 2014, **9**, e108978.

12 Y. T. Liu, H. Zhang, H. Wang, C. L. Tao, G. Q. Bi and Z. H. Zhou, *Nat. Commun.*, 2022, **13**, 1–17.
